# Core Health Outcomes in Childhood Epilepsy (CHOICE): Development of a core outcome set using systematic review methods and a Delphi survey consensus

**DOI:** 10.1111/epi.14735

**Published:** 2019-04-25

**Authors:** Holly Crudgington, Morwenna Rogers, Lucy Bray, Bernie Carter, Janet Currier, Colin Dunkley, Frances M. Gibbon, Dyfrig Hughes, Samantha Lyle, Deborah Roberts, Catrin Tudur Smith, Paul Gringras, Deb K. Pal, Christopher Morris

**Affiliations:** ^1^ Basic and Clinical Neuroscience Department Institute of Psychiatry, Psychology, and Neuroscience King's College London London UK; ^2^ University of Exeter Medical School, College of Medicine and Health University of Exeter Exeter UK; ^3^ Faculty of Health and Social Care Edge Hill University Ormskirk UK; ^4^ Lay coinvestigator and epilepsy services user London UK; ^5^ Sherwood Forest Hospitals National Health Service Foundation Trust Sutton‐in‐Ashfield UK; ^6^ Noah's Ark Children's Hospital for Wales Cardiff and Vale University Health Board Cardiff UK; ^7^ Centre for Health Economics and Medicines Evaluation Bangor University Bangor UK; ^8^ Department of Biostatistics University of Liverpool Liverpool UK; ^9^ Evelina London Children's Hospital London UK; ^10^ Medical Research Council Centre for Neurodevelopmental Disorders King's College London London UK; ^11^ King's College Hospital London UK

**Keywords:** children, core outcome set, epilepsy, pediatric, young people

## Abstract

**Objective:**

Establishing a core set of outcomes to be evaluated and reported in intervention trials aims to improve the usefulness of health research. There is no established core outcome set (COS) for childhood epilepsies. The aim of this study was to select a COS to be used in evaluative research of interventions for children with rolandic epilepsy (RE).

**Methods:**

We followed guidance from the COMET (Core Outcome Measures in Effectiveness Trials) Initiative. First, we identified outcomes that had been measured in research through a systematic review. Second, young people with RE, parents, and professionals were invited to take part in a Delphi survey in which participants rated the importance of candidate outcomes. Last, a face‐to‐face meeting was convened to seek consensus on which outcomes were critical to include and to ratify the final COS.

**Results:**

From 37 eligible papers in the review, we identified and included 48 candidate outcomes in the survey. We sent invitations to 165 people registered to take part in the survey; of these, 102 (62%) completed Round 1, and 80 (78%) completed Round 2 (three young people, 16 parents, 61 professionals). In Round 2 we included four additional outcomes suggested by participants in Round 1. The consensus meeting included two young people, four parents, and nine professionals who were eligible to vote and ratified the COS as 39 outcomes across 10 domains.

**Significance:**

Our methodology was a proportionate and pragmatic approach toward producing a COS for evaluating research on interventions aiming to improve the health of children with RE.

**Key Points**

- There was no established core outcome set for childhood epilepsy
- Consensus‐based methods were used to rate the importance of different outcomes in rolandic epilepsy; this included two rounds of a Delphi survey and a face‐to‐face meeting that included young people with rolandic epilepsy, parents, and various professionals
- We identified 39 outcomes across 10 domains that contributed toward a core outcome set for use in epilepsy research

## 1 INTRODUCTION

Epilepsy is a common neurological disorder that can be defined by a persisting tendency for epileptic seizures. Epilepsy encompasses many different conditions, including around 30 different epilepsy syndromes and affects people of all ages, including children.^1^ Seizure reduction, freedom from seizures, and a significant reduction in duration and intensity of seizures are typical primary outcomes in trials evaluating interventions for epilepsy. However, it is important to consider the adverse effects of antiepileptic medication as well as nonseizure outcomes, particularly in developing children.

The social and psychological consequences of seizures and children's perspectives are becoming more valued, and health‐related quality of life (HRQoL) is an increasing focus for research.^2^ It is important to consider that epilepsy‐specific quality of life is not determined by seizures alone but can also be influenced by the child's learning, mental health, and social support.^3^, ^4^ To overcome these issues, it is crucial to decide on a core set of outcomes that are of greater importance to children and their families.

The variety of outcomes assessed in research and the different ways outcomes are measured can reduce the ability to combine and compare studies.^5^ Recognizing a core set of outcomes to be measured and reported in all trials of interventions for specific conditions aims to advance the usefulness of research and avoid waste.^6^, ^7^, ^8^ A core outcome set (COS) that recommends the same suite of outcomes measured in the same way reduces both heterogeneity between studies and outcome reporting bias. It can also increase the potential for carrying out meta‐analysis for important outcomes. The development of a COS should include the views of patients, carers, and health professionals.^7^ A COS may also be useful for other types of research, clinical audit, and structuring routinely collected health services clinical data. A COS specifies which aspects of health are to be assessed and how to measure them.

Currently, there is no COS for evaluative research of interventions in children with epilepsy. The Core Outcome Measures in Effectiveness Trials (COMET) Initiative database recently added a study focused on West syndrome^9^ and a study conducted in Sri Lanka on the development of outcome criteria to measure effectiveness of antiepileptic medication.^10^ The National Institute for Health Care and Excellence guidelines recommend seizure freedom as a primary outcome alongside seizure reduction, quality of life, and cognitive functioning as secondary outcomes.^11^ Cochrane reviewers advise focusing on longer‐term outcomes such as psychosocial and health economic outcomes.^12^ Scottish guidance recommends including aspects of academic attainment and mental health outcomes^13^ and the International League Against Epilepsy (ILAE) has published guidance on outcome measurement for clinical trials.^14^, ^15^ Children are included in the Common Data Elements recommended for epilepsy research by the National Institute of Neurological Disorders and Stroke (NINDS).^16^ The NINDS recommends a comprehensive list of items across various domains, but children and parents were not consulted in the process.^16^

The aim of this study was to develop a COS relevant to evaluative research on interventions for children with rolandic epilepsy (RE), as an exemplar of common childhood epilepsy syndromes. RE is also known as “childhood epilepsy with centrotemporal spikes” in the revised ILAE classification.^1^ RE is the most common childhood epilepsy, affecting 17%‐25% of children with epilepsy in the 5‐ to 14‐year age range.^17^, ^18^, ^19^ The syndrome is associated with specific neuropsychological impairments such as in speech and language, literacy, and attention as well as motor coordination deficits but is not associated with autism spectrum disorder or intellectual disability.^20^, ^21^, ^22^ Our study focused on children of school age (5‐16 years old) with RE, and our protocol is published.^23^ Specifically, our objectives were to review published research to identify outcomes reported in research and to seek consensus on which outcomes were perceived to be most important to measure in research. The work was conducted in partnership with families, health professionals, and epilepsy charities in the UK.

Our work is motivated by the necessity to change the agenda from a seizure‐centered medical model toward broader child and family priorities and to focus scarce resources on the most important outcomes.^24^ Our primary aim was to propose a COS for evaluative trials, but the findings may also inform decisions on outcomes measured in audits and/or routinely collected services data. The scope of this study included outcomes of any medical or social intervention where the aim was to improve the health of children with epilepsy and was not limited to medication. This study is part of a program of work aiming to improve broad HRQoL for children with epilepsy. The COS will inform decisions about outcomes to be measured in a future clinical trial evaluating interventions for RE scheduled to begin recruitment in 2019.

## 2 MATERIALS AND METHODS

### 2.1 Ethics and registration

The study was conducted in line with COMET methodological recommendations,^25^ with a published protocol^23^ and was registered on the COMET database (www.comet-initiative.org/studies/details/1030). Our study was approved by the National Health Service (NHS) Health Research Authority (North East–Newcastle & North Tyneside 1 Research Ethics Committee, reference 18/NE/0014). Participants registered for the Delphi survey through our website (www.castlestudy.org.uk). Taking part in the Delphi was regarded as implicit consent. Young people took part in the Delphi if their parents agreed and provided them with the online Delphi link. Written consent was gathered at the face‐to‐face consensus meeting for parents, young people, and professionals. The study is reported in line with COS‐STAR (Core Outcome Set–Standards for Reporting) guidance^26^ and the GRIPP2 short form for Patient and Public Involvement (PPI)^27^ and the review is reported with reference to PRISMA (Preferred Reporting Items for Systematic Reviews and Meta‐Analyses).^28^

To develop the COS, we undertook three steps: (1) identifying candidate outcomes, (2) rating the importance of candidate outcomes in a two‐round Delphi survey, and (3) a face‐to‐face consensus meeting to ratify results of the Delphi survey and agree core outcomes. We convened an advisory panel (AP) of young people with epilepsy and their carers alongside to consult on various decisions throughout each step.

#### 2.1.1 Step 1. Identifying candidate outcomes

We identified candidate outcomes via structured, systematic review methods described in our protocol.^23^ Briefly, we looked for (1) primary evaluations and systematic reviews of interventions for RE, (2) qualitative or mixed methods studies about experiences and preferences for outcomes, and (3) epilepsy‐specific and generic patient‐reported outcome measures used with children with epilepsy. We searched for systematic reviews using terms for RE or childhood epilepsy in the Cochrane Database of Systematic Reviews, MEDLINE (via OvidSP), Embase (via OvidSP), PsycINFO (via OvidSP), and CINAHL (via EBSCOhost). We searched for controlled trials via the CENTRAL database and checked previously found systematic reviews for additional relevant trials. We also searched the World Health Organization International Clinical Trials Registry Platform for ongoing trials. Finally, we searched for qualitative or mixed methods research on MEDLINE, CINAHL, and PsycINFO using terms for epilepsy combined with terms for qualitative research, experience, and HRQoL. The electronic search was carried out in October 2017 by M.R. (Appendix S1). H.C. reviewed and screened the abstracts and selected references. Decisions on eligibility where there was uncertainty were made in consultation with two other reviewers (C.M. and D.K.P.). As outlined in our protocol,^23^ two people did not screen the references independently, because the additional resources were not justified by the risk of missing outcome domains, as we expected considerable duplication. H.C. coded outcomes extracted from the papers in consultation with C.M. using the COMET taxonomy^29^; any doubts about coding were resolved in consultation with the wider team. H.C. extracted and entered information from the papers into spreadsheets, including population, outcomes, measurement instruments, and any other salient information, and then this summarized in table form (Appendix S2). We did not assess risk of bias for included studies, as this was not relevant to the aim of this study.

#### 2.1.2 Step 2. Rating the importance of outcome domains in a two‐round Delphi survey

The outcome domains identified in step 1 were taken forward for importance rating in a Delphi survey. We conducted the online Delphi survey over two rounds (R1 and R2) using DelphiManager software.^30^ Our protocol proposed three rounds, but we shortened this to two rounds to reduce potential attrition and mitigate time constraints. We recruited participants from three key stakeholder groups: young people with RE aged 7‐16 years, parents of children with RE, and professionals working with this group of children (pediatricians, pediatric neurologists, epilepsy specialist nurses, etc). We recruited participants through various platforms, including epilepsy charities, professional societies, and regional networks via OPEN UK (Organisation of Paediatric Epilepsy Networks). We posted advertisements on social media platforms (eg, Facebook and Twitter). Four NHS hospitals were set up as participant identification centers to enable clinicians to recruit patients, parents, and colleagues. We directed interested participants to the study website, where they could register using an online form.

Participants were asked to rate the importance of each outcome in the Delphi survey using a scale from 1 to 9 in which options 1‐3 were labeled “less important,” options 4‐6 were “important but not critical,” and options 7‐9 indicated “critical for inclusion” in the COS. Participants were able to suggest additional outcomes in R1, which were considered by the core team members for inclusion in R2 (Appendix S4). We considered whether the suggested outcomes were actually different from the concepts already covered in the existing Delphi and whether they had been suggested by more than one person. In R2, participants were shown the distribution of other stakeholders’ scores from R1 in histograms as well as their own R1 score. They were asked to use this information to reflect on their score and rate the outcome again. Participants were able to give reasons for changing their score and leave free text comments.

We sent the Delphi survey link to people who registered interest online with a valid email address. R1 and R2 were open for 2 weeks each with a 1‐week interval in between. After R2 closed, we downloaded the participant data and converted them into the percentage of stakeholders scoring from 1 to 9 across all outcomes. Our predefined consensus criteria were (1) most important “core” outcomes agreed by most stakeholders (>70% in each stakeholder group rated 7‐9), (2) less important outcomes (>70% in each stakeholder group rated 1‐3), and (3) those where there was partial or no agreement.

#### 2.1.3 Step 3: Consensus meeting

Results from R2 of the online Delphi (Table 3) were shown at a face‐to‐face half‐day meeting in London. We sent invitations to participants who had completed both R1 and R2 of the Delphi. We encouraged parents to bring their children to the meeting if their child had also taken part in the Delphi. Travel costs were reimbursed on behalf of participants as well as a payment given to nonsalaried individuals. All three stakeholder groups were represented at the meeting (Appendix S5). A member of the research team (C.M.) chaired the meeting, and ground rules were agreed to ensure that all participants felt comfortable about speaking out in the group.

Outcomes that had met the a priori criteria of “consensus in” from the Delphi were initially displayed; all participants agreed that no further discussion was needed about their inclusion in the COS. All the remaining “no consensus” outcomes were displayed and discussed at the meeting. We gave participants red and green cards to vote with. Holding up a red card meant that the outcome was not important enough to include in the COS. Holding up a green card meant that the participant thought it critical that the outcome be included. The chair ensured that contrasting views about voting were discussed and that equal opportunity was given to participants to discuss their voting decisions. Outcomes meeting the criteria for “consensus in” during the meeting were incorporated into the COS. The final COS was presented and ratified to the group via email after the meeting so that people could have further time to think about their decisions and confirm.

### 2.2 Patient and public involvement

Two parents of children with epilepsy were coapplicants when we sought funding for the program of research within this nested study and are coinvestigators. A Family Engagement Officer (FEO) convened an AP in the south of England to involve young people and parents as meaningful partners in the development and implementation of our research. The FEOs recruited young people with epilepsy and their parents through various UK charities (Young Epilepsy, Epilepsy Action, Epilepsy Research UK), clinical networks (including consultant clinics and epilepsy specialist nurses), word‐of‐mouth, online parent forums, and social media groups. We consulted AP members through face‐to‐face meetings and also remotely using email and telephone. The AP members were involved in reviewing the Core Health Outcomes in Childhood Epilepsy (CHOICE) documents sent to the ethics committee and the Delphi survey. Members of the AP were asked to consider ease of the instructions and use of the survey.

AP members provided insight as to the ease of the Delphi survey and the relevance of outcomes. Modifications were made to the Delphi survey based on AP feedback, and some wording for Delphi instructions were changed. Two parent lay coapplicants (D.R., J.C.) were part of the consensus meeting alongside the southern England FEO who helped to facilitate the contribution of parents and children in the meeting.

## 3 RESULTS

### 3.1 Step 1: Identifying candidate outcomes

**Figure 1:**
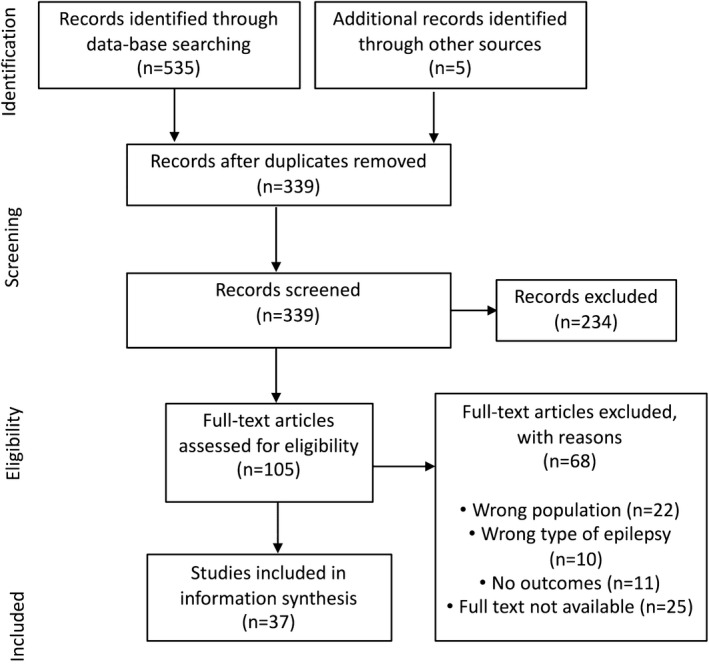
PRISMA (Preferred Reporting Items for Systematic Reviews and Meta‐Analyses) flowchart of literature review

Thirty‐seven papers were included in the review (Figure 1); 181 outcomes were recorded verbatim. A provisional list of 177 outcomes (Appendix S2) was reviewed at a face‐to‐face meeting. There were a large number of outcomes that overlapped considerably, so outcomes were coded using the COMET taxonomy^29^ into the following domains: physiological nervous system outcomes, physical functioning, social functioning, role functioning, emotional functioning/well‐being, cognitive functioning, global quality of life, and adverse events/effects. Similar outcomes were discussed and aggregated at the meeting that resulted in 48 overall outcomes for inclusion in R1 of the Delphi survey (Table 1). Each outcome was given a lay domain name and description for use in the survey. The descriptions were agreed upon with two parent lay coapplicants at the study meeting.

**Table 1:** Outcomes used in R1 & R2 of the Delphi survey

Outcome ID and name in Delphi	Description	Domain name
1. Seizure freedom	Not having seizures	Seizures
2. Seizure frequency	How often seizures occur	Seizures
3. Seizure duration	How long a seizure lasts	Seizures
4. Seizure severity	How bad seizures are in terms of effects on the person during and after seizures, such as falls or injuries, incontinence, confusion, and time to recover afterward	Seizures
5. Time to fall asleep	Time it takes to fall asleep from snuggling down	Sleep
6. Time spent asleep	Total time spent asleep each day	Sleep
7. Awakenings	Waking in the night that parents/carers are aware of	Sleep
8. Breathing difficulties during sleep	Might include snoring or gasping for breath	Sleep
9. Daytime sleepiness	Feeling sleepy or actually sleeping during the day	Sleep
10. Fatigue	Lacking in energy	Physical functioning
11. Pain	Unpleasant physical sensation	Physical functioning
12. Coordination and balance	Using parts of the body together and efficiently, such as to ride a bike, stand on one leg, catch and throw	Physical functioning
13. Movement ability	Walking, running, jumping, hopping	Physical functioning
14. Manual ability	Dexterity in handling objects, handwriting	Physical functioning
15. Self‐care	Daily routines such as eating, washing and dressing, toileting	Usual activities
16. Ability to join in activities with others	Joining in with people, such as playing out withfriends, doing sports, joining in things	Social functioning
17. Ability to play on one's own	Reading, computer, imaginary play	Social functioning
18. Friendships	Forming and maintaining friendships	Social functioning
19. Engagement in school life	Feeling part of the school community	Social functioning
20. Social life	Engagement with friends and peers, such as going out, sleepovers, cinema	Social functioning
21. Experience of people's attitudes toward epilepsy	Bullying, social exclusion	Social functioning
22. Behaving appropriately	Being able to control emotions and respond to situations in context	Behavior
23. Impulsivity	Acting without thinking	Behavior
24. Fidgeting	Restless, being on the go, moving or squirming	Behavior
25. Feeling normal	Feeling like other people of the same age	Mental health
26. Feelings about having epilepsy	Emotions or reactions to having epilepsy, such as embarrassment, shame, stigma	Mental health
27. Happiness	Feeling or showing pleasure or contentment	Mental health
28. Sadness	Feeling or showing sorrow or being unhappy	Mental health
29. Worried	Being anxious or troubled about actual or potential problems	Mental health
30. Annoyed	Being slightly angry or irritated	Mental health
31. Self‐esteem	Overall feelings about yourself	Mental health
32. Mood swings	Quick unexplained changes of mood	Mental health
33. Self‐harm	Thinking about hurting yourself on purpose or wishing you were dead	Mental health
34. Concealment	Not telling people about epilepsy	Mental health
35. Fears of having a seizure	Having a seizure in public, being injured during a seizure, dying during a seizure, what other people will do during a seizure	Mental health
36. Literacy	Reading, writing, spelling	Cognition
37. Speech and language	Making yourself understood and understanding when spoken to	Cognition
38. Memory	Short and long term	Cognition
39. Concentration	Focusing on something for the required period of time	Cognition
40. Learning	Gaining new skills and knowledge generally	Cognition
41. School attendance	Attending school and engaging in school curriculum	Cognition
42. Academic attainment	Reaching personal potential through studying and completing assigned tasks and projects, and advancing to next stages of education	Cognition
43. Executive functioning	The ability to plan and organize activities; executive functions help you manage life tasks of all types; for example, executive functions let you organize a trip, a research project, or a paper for school effectively	Cognition
44. Overall quality of life	How you feel your life is generally	Global quality of life
45. Adverse events	Any unintended effects of treatments, side effects	Adverse events
46. Relationships with parents and siblings	Getting along well with and feeling close to other members of family	Family functioning
47. Family life	Impact of epilepsy on family life such as parent work opportunities and/or leisure time	Family functioning
48. Parent health	Parent's physical and emotional well‐being	Family functioning
Outcomes suggested and included in R2		
49. Unplanned epilepsy‐related admissions to hospital as an inpatient	Unexpectedly needing to be admitted to hospital	Adverse events
50. Unplanned hospital attendances at Accident and Emergency Department	Visiting the hospital due to an acute medical emergency	Adverse events
51. Attendance for medical appointments in outpatients	Routine attendances for medical epilepsy management	Seizures
52. Drug treatment failure (adverse events or poor seizure control)	Stopping medication because it is not working or causing problems	Adverse events

### 3.2 Step 2: Delphi survey

One hundred sixty‐five people registered interest through our study website. Of the 165 interested people, 102 participants took part in R1 (professionals, n = 76, 75%; parents, n = 23, 20%; young people, n = 3, 3%), and 80 from R1 completed R2 (professionals, n = 61, 76%; parents, n = 16, 20%; young people, n = 3, 4%; Table 2). The majority of people who completed R2 were from London (professionals, 30%; parents and young people, 21%), with full demographics of participants available in Appendix S3. One professional withdrew from the study in R1 due to work commitments. Four people did not fully answer R1 questions, and only the questions they answered were included in the analysis. Twenty‐two people did not fully complete R2 questions despite logging in, and only the questions they answered were analyzed. R1 and R2 of the Delphi were open for 2 weeks, with a 1‐week gap in between the rounds to allow the histograms to be created and uploaded for R2. Forty‐eight outcomes were rated in R1 (Table 1) and an additional 19 outcomes were suggested in R1, of which four were brought forward based on predefined decisions (Appendix S4). After the close of the Delphi survey, 11 outcomes met the a priori condition for “consensus in” from R2. Delphi R1 and R2 scores are shown in Table 3. The attrition rate from R1 to R2 was 22% overall (33% of parents, 20% of professionals, 0% of young people), displayed in Table 2.

**Table 2:** Response of R1 and R2 of the Delphi survey

Stakeholder group	Registered interest, n	Round 1, n (% who were eligible to take part)	Round 2, n (% who were eligible to take part)
Professionals total	120	76 (63)	61 (80)
Pediatricians	51	33 (65)	26 (82)
Pediatric neurologists	16	14 (88)	12 (86)
Epilepsy nurses	22	15 (68)	12 (87)
Consultant in sleep medicine	6	3 (50)	2 (67)
Physiologists	5	4 (80)	3 (75)
Respiratory and sleep physiologists	11	3 (27)	2 (67)
Dietetics lecturer	1	1 (100)	1 (100)
NHS manager	2	2 (100)	2 (100)
Child and adolescent psychiatrist	1	1 (100)	1 (100)
CEO of children's charity	1	0	0
Child health lecturer	1	0	0
Clinical psychologist	1	0	0
Neuropsychologist	2	0	0
Parents	40	23 (58)	16 (67)
Young people	5	3 (60)	3 (100)
Total	165	102 (62)	80 (78)

Note

One professional withdrew. Four people did not answer fully in R1. Twenty‐two people did not fully answer in R2.

Abbreviation: NHS, National Health Service.

**Table 3:** R1 & R2 percentage of scores for 7‐9 (critical for inclusion) across all three stakeholder groups

Outcome	Round 1			Round 2		
	Professionals, n = 76	Parents, n = 23	Young people, n = 3	Professionals, n = 61	Parents, n = 16	Young people, n = 3
1. Seizure freedom						
	85%	83%	67%	94%	88%	67%
2. Seizure frequency						
	91%	91%	67%	95%	94%	100%
3. Seizure duration						
	63%	87%	67%	73%	94%	100%
4. Seizure severity						
	77%	87%	33%	89%	100%	33%
5. Time to fall asleep						
	35%	22%	0%	19%	35%	0%
6. Time spent asleep						
	55%	39%	0%	48%	71%	0%
7. Waking from sleep						
	59%	39%	67%	55%	76%	67%
8. Breathing difficulties during sleep						
	54%	65%	67%	55%	75%	67%
9. Daytime sleepiness						
	65%	39%	67%	73%	47%	67%
10. Fatigue						
	55%	35%	33%	52%	53%	33%
11. Pain						
	37%	57%	0%	26%	56%	0%
12. Coordination and balance						
	40%	52%	100%	41%	59%	100%
13. Movement ability						
	27%	30%	67%	26%	31%	67%
14. Manual ability						
	31%	35%	33%	26%	47%	33%
15. Self‐care						
	42%	30%	33%	28%	41%	33%
16. Ability to join in activities with others						
	59%	48%	67%	64%	59%	67%
17. Ability to play on one's own						
	45%	26%	67%	36%	35%	67%
18. Friendships						
	58%	52%	67%	62%	53%	67%
19. Engagement in school life						
	74%	57%	67%	75%	59%	67%
20. Social life						
	64%	52%	33%	67%	65%	33%
21. Experience of other people's attitudes toward epilepsy						
	49%	43%	67%	46%	50%	67%
22. Behaving appropriately						
	56%	61%	67%	57%	71%	67%
23. Impulsivity						
	42%	48%	67%	46%	65%	100%
24. Fidgeting						
	38%	43%	33%	38%	65%	33%
25. Feeling normal						
	68%	61%	33%	79%	65%	33%
26. Feelings about having epilepsy						
	68%	74%	33%	70%	65%	33%
27. Happiness						
	67%	64%	100%	79%	65%	100%
28. Sadness						
	65%	61%	33%	66%	63%	33%
29. Worried						
	67%	57%	67%	67%	63%	67%
30. Annoyed						
	49%	52%	100%	46%	69%	100%
31. Self‐esteem						
	69%	65%	67%	77%	69%	100%
32. Mood swings						
	60%	52%	67%	54%	69%	100%
33. Self‐harm						
	68%	59%	50%	70%	75%	67%
34. Concealment						
	57%	52%	33%	49%	56%	33%
35. Fears of having a seizure						
	74%	74%	100%	84%	81%	100%
36. Literacy						
	57%	57%	67%	66%	81%	67%
37. Speech and language						
	66%	57%	33%	67%	69%	33%
38. Memory						
	72%	65%	67%	72%	81%	67%
39. Concentration						
	72%	65%	100%	79%	81%	100%
40. Learning						
	79%	74%	100%	80%	94%	100%
41. School attendance						
	70%	48%	67%	77%	50%	67%
42. Academic attainment						
	63%	52%	67%	72%	69%	100%
43. Executive functioning						
	63%	48%	100%	67%	69%	100%
44. Overall quality of life						
	92%	74%	67%	93%	88%	67%
45. Adverse events or reactions						
	71%	78%	67%	72%	81%	67%
46. Relationships with parents and siblings						
	58%	48%	100%	61%	63%	100%
47. Family life						
	62%	48%	67%	64%	56%	67%
48. Parental health						
	51%	43%	67%	46%	50%	67%
49. Unplanned epilepsy‐related admissions to hospital as inpatient						
				70%	67%	67%
50. Unplanned hospital attendances at Accident and Emergency Department						
				70%	64%	33%
51. Attendance for medical appointments in outpatients						
				33%	44%	0%
52. Drug treatment failure (adverse events or poor seizure control)						
				78%	87%	100%

Note

Green highlight indicates >70% of participants rated as 7‐9 (critical for inclusion). Yellow highlight indicates >50% of participants rated as 7‐9 (critical for inclusion).

### 3.3 Step 3: Consensus meeting

**Figure 2:**
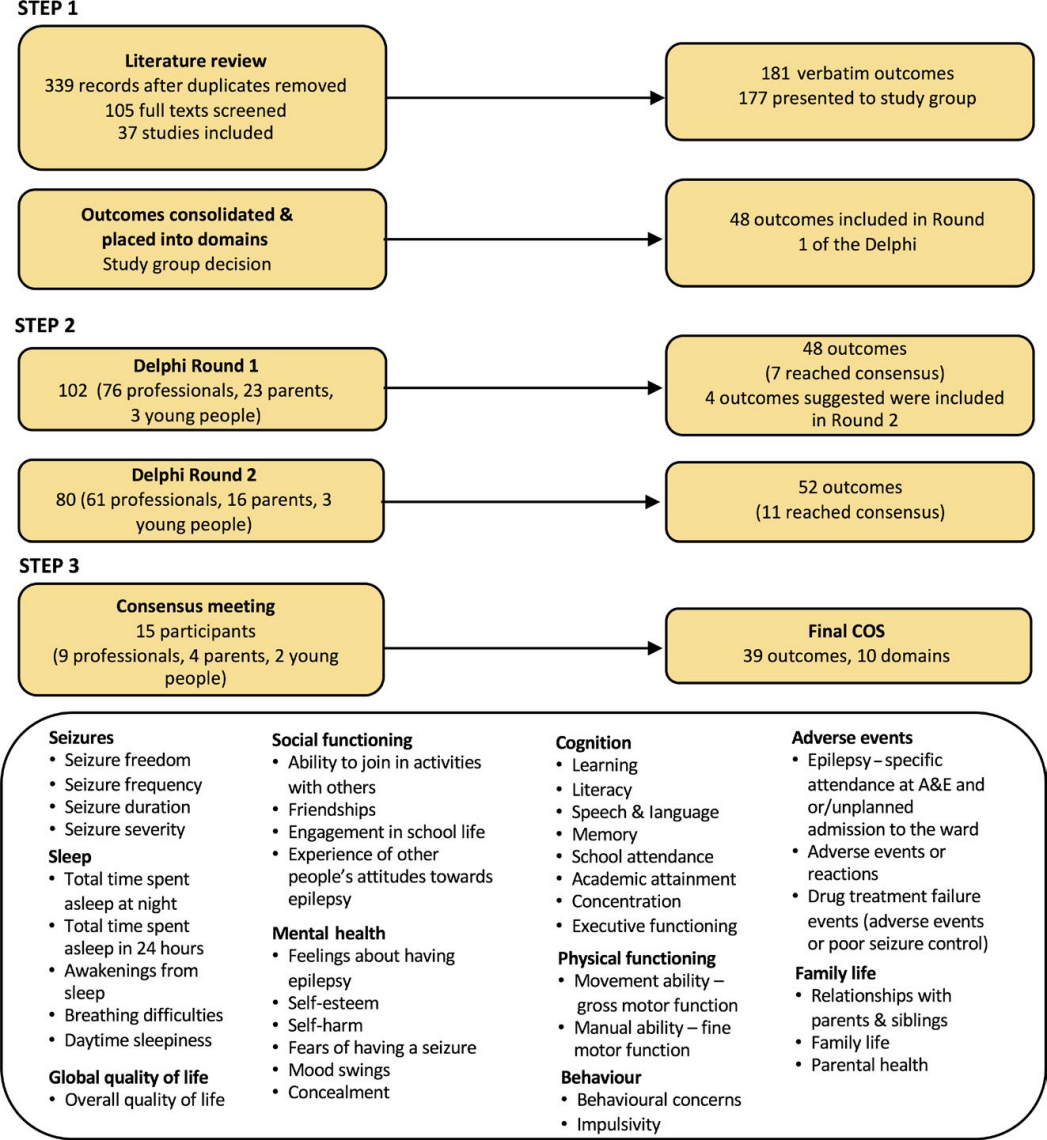
Overview of core outcome set (COS) development and final COS. Abbreviation: A&E, Accident and Emergency Department

Nineteen people were present at the face‐to‐face consensus meeting, and 15 were eligible to vote: two young people (aged 11 and 12 years), four parents, and nine professionals (two pediatricians, two pediatric neurologists, two sleep consultants, one clinical psychologist, one physiologist, and one professor of children's nursing), with information about meeting members available in Appendix S5. Five of the voters had not taken part in the Delphi survey but were deemed eligible as they had sufficient knowledge of the CHOICE project. Twenty‐eight outcomes were voted as critical for the COS from the 41 no‐consensus outcomes. Overall, 39 outcomes were deemed critical for inclusion in the COS split into 10 domains: seizures, sleep, global quality of life, mental health, social functioning, physical functioning, cognition, behavior, family life, and adverse events. An overview of the final COS and its development process is shown in Figure 2 and the results of the consensus meeting in Table 4.

**Table 4:** Summary of consensus meeting voting results

Outcome	Number of stakeholder groups (of 3) achieving consensus prior to meeting	% of 15 meeting participants voting as critical for inclusion in COS	% of meeting participants voting as not critical	Category of meeting conclusion
Seizure freedom	3	100	0	1
Seizure frequency	3	100	0	1
Seizure duration	3	100	0	1
Seizure severity	2	100	0	2
Time to fall asleep	0	0	100	4
Time spent asleep in 24 hours	1	100	0	3
Time spent asleep each night	1	100	0	3
Awakenings from sleep	2	100	0	2
Breathing difficulties during sleep	2	93	6	2
Daytime sleepiness	2	93	6	2
Fatigue	0	0	100	4
Pain	0	0	100	4
Movement ability–gross motor function	1	100	0	3
Manual ability–fine motor function	1	93	6	3
Self‐care	0	0	100	4
Ability to join in activities with others	1	100	0	2
Ability to play on one's own	1	0	100	4
Friendships	1	93	7	2
Engagement in school life	2	100	0	2
Experience of other people's attitudes toward epilepsy	1	100	0	2
Behavioral concerns	2	100	0	3
Impulsivity	1	79	21	2
Fidgeting	0	0	100	4
Feelings about having epilepsy	2	100	0	3
Self‐harm	3	100	0	1
Fears of having a seizure	3	100	0	1
Self‐esteem	2	100	0	2
Mood swings	1	100	0	2
Concealment	0	100	0	2
Learning	3	100	0	1
Concentration	3	100	0	1
Literacy	2	100	0	2
Memory	3	100	0	1
Speech and language	0	93	7	2
School attendance	2	100	0	2
Academic attainment	2	100	0	2
Executive functioning	1	100	0	2
Relationships with parents and siblings	1	100	0	2
Family life	1	100	0	2
Parental health	1	100	0	2
Overall quality of life	3	100	0	1
Adverse events or reactions	3	100	0	1
Drug treatment failure events (adverse events or poor seizure control)	3	100		1
Epilepsy specific attendance at A&E and/or unplanned admission to the ward	2	100	0	3

Note

Outcomes have been categories based on the following: (1) item previously “consensus in” and no discussion needed, (2) discussed and voted in, (3) discussed and agreed to combine with another outcome/word differently (or to be considered as part of how an outcome is measured), (4) agreed not to discuss further or voted as “not critical for the COS.”

Abbreviation: COS, core outcome set.

## 4 DISCUSSION

This study enabled young people with epilepsy, parents, and health professionals from different backgrounds to come together and reach consensus on important outcomes to measure in evaluative research on RE. The 39 outcomes included in the COS were rated as “critical” by >70% of people in all three stakeholder groups. Using Delphi methodology avoids potential overinfluence of one type of stakeholder and captures different perspectives. Hence, our COS represents the view shared by young people with epilepsy, parents, and various health professionals working with children with epilepsy. Future research evaluating interventions for children with RE should use the CHOICE COS as a reference for selecting outcomes and consider its adaptability for other childhood epilepsies.

The CHOICE COS is the result of a transparent process that was inclusive of young people and parents, as well as professionals in the field of epilepsy. The 39 outcomes across 10 domains perhaps represent more of a “comprehensive” rather than a “core” outcome set (Figure 2). A COS is meant to be a minimum set of outcomes to report. Further work could consider reducing the number of outcomes by ranking which outcomes are of most importance, relevant to each other. We did not have enough time during our face‐to‐face meeting to undertake a ranking task. The COMET handbook^25^ identifies examples of where ranking has been used^31^, ^32^, ^33^ as well as a recent study from the COMET database conducted in Sri Lanka that used a ranking method.^10^

Our COS captured commonly reported items such as “seizure frequency” consistent with existing guidelines.^11^, ^12^, ^13^, ^14^, ^15^, ^16^ However, in contrast, our COS highlights non–seizure‐related outcomes such as “school attendance” (attending school and engaging in school curriculum) and “feelings about epilepsy” (emotions or reactions to having epilepsy such as embarrassment or stigma) agreed as critical for inclusion in the COS. All COS outcomes and their definitions are in Table 1. Outcomes such as “pain” were not deemed as important for an epilepsy COS across the stakeholder groups. This might be a reflection of the relevance of that outcome for this specific type of epilepsy.^21^, ^34^ In the consensus meeting, the young people involved were vocal and fairly represented, and their views were often persuasive on other participants. The inclusion of more child‐centered outcomes suggests that the seizure‐centered view is not the only important outcome for HRQoL in young people with RE. Pragmatically, to inform our trial we are identifying and assessing epilepsy‐specific HRQoL measures.

The COMET database included a study conducted in Sri Lanka that has developed outcome criteria to measure effectiveness of antiepileptic therapy in children, which included young people with epilepsy as one of the stakeholder groups.^10^ The study was published while we were in the process of conducting our study. Their study recruited 15 young people with epilepsy, and the outcomes that reached consensus were very similar to ours, which adds assurance that our COS captures outcomes important to young people with epilepsy across different settings. For example, their work included outcomes such as frequency of seizures, severity of seizures, seizure freedom, cognitive function, activities that children like to do, school attendance, behavior, and quality of life, which map well onto the COS we propose. Interestingly, they ranked their outcomes with frequency of seizures being most important, followed by quality of life, which might suggest that seizures affect QoL.^10^

Major strengths of the CHOICE study include a prior defined protocol,^23^ following COMET initiative methodology and using the standardized COMET taxonomy.^29^ The DelphiManager software ensured that the views of all three stakeholder groups were given equal representation despite varying numbers of participants in each group. The DelphiManager survey method ensures fair representation, as analysis is assessed within stakeholder group before comparing across stakeholder groups. This is the same method we used in the consensus meeting, as we used proportions of stakeholder type to balance representation and compared within stakeholder group before comparing across groups. We included the views of young people with RE, their parents, and professionals in the Delphi, and we convened APs alongside to ensure PPI input at both the development and the implementation stage of the COS.

A potential limitation of our study is that we conducted a proportionate rather than a comprehensive systematic review. Systematic reviews are time‐consuming and for the purposes of COS development they may not generate additional outcomes for conditions that are common.^25^ We did use various databases for the review and included a wide range of studies^23^; we also provided opportunities for AP and survey participants to suggest any outcome domains not identified in the review. It is evident from the review results that a large number of varied outcomes have been used in epilepsy research, which demonstrates the important challenge of developing an agreed‐upon COS. We encountered difficulties in recruiting RE patients to the CHOICE Delphi study, particularly that the term “rolandic” was unfamiliar to many families. Our participant information sheets and adverts used rolandic as well as the ILAE term “childhood epilepsy with centrotemporal spikes.”

The CHOICE study is a work package within a larger program of work called “Changing Agendas on Sleep Treatment and Learning” (CASTLE; http://castlestudy.org.uk), and decisions were made to reduce to two rounds rather than three in the Delphi survey to deliver the COS in time to inform design of the clinical trial. The impact of this meant that participant burden was lessened, which perhaps is a reason we had little attrition between the two rounds. However, a three‐round Delphi would have possibly meant a larger sample of people may have reached consensus on more items.

The number of participants in our survey, particularly young people, was low despite our varied approach to recruitment. However, the three young people who took part in R1 of the survey also took part in R2. Attrition rates overall were good for the survey, with 78% of those who took part in R1 taking part in R2. This was despite the survey only being open for 2 weeks for each round. However, even with multiple email reminders, some telephone calls were needed to improve the response rate. Clinical work load and school term time, as well as the short window that the rounds were open for, are likely to have contributed to the time it took for participants to respond. The number of people in each stakeholder group who were able to attend the consensus meeting after participating in the online Delphi was lower than expected based on the number invited. The meeting was held on a weekday during school time, and parents and health professionals may have had different preferences for the timing of the meeting; specific needs such as clinic times for professionals and childcare needs should be taken into consideration for future studies.

The analysis of the Delphi results grouped professionals into one stakeholder group. We decided to group professionals due to small numbers in some professions. However, the varying roles of stakeholders may have had some influence on the level of engagement with a COS of children with RE. For example, the majority of professionals were epilepsy specialist nurses, pediatric neurologists, and pediatricians, and the smaller numbers were seen in lecturers and NHS managers. Voting in the consensus meeting was not conducted anonymously, because we wanted to seek consensus by having active discussion about shared or differing opinions and for participants to consider how other stakeholders voted.

The CHOICE COS focused principally on RE as an exemplar of childhood epilepsy. Focusing on RE avoided preferences for outcomes that might be affected by including children who have associated conditions such as autism or cerebral palsy. However, we suggest that the CHOICE COS could potentially be generalized across other childhood epilepsies. Future work could consider the extent to which any variations might be necessary to validate the COS in other childhood epilepsy syndromes. The scope of our work was primarily UK‐based, but the COS may have broader international relevance. To promote uptake of the suggested COS internationally, an international consensus would be needed.

Having decided which outcomes to measure, the next step in the COS process is to decide how best to measure each of these outcomes and how to define them using published guidance.^35^ The time burden for research participants will need consideration for the comprehensive outcome list we currently propose. Although further work is necessary to reduce the COS and define the outcomes further, our next step will be to identify and assess the measurement properties of epilepsy‐specific HRQoL measures to inform the CASTLE trial and assess whether the outcomes we propose are measured by these instruments. We will consult guidelines on the selection of outcome measurement instruments for a COS developer by COSMIN (Consensus‐Based Standards for the Selection of Health Measurement Instruments).^35^

## 5 CONCLUSIONS

We recommend that future evaluative research in RE considers utilizing the CHOICE COS as a framework for selecting outcomes for evaluative research**.** The CHOICE COS is a fair representation of the views of young people with RE, their parents, and professionals and has used established methodology.^25^ Further work to reduce the COS to a smaller number of outcomes by ranking will make the COS more manageable. However, we propose that our work toward a COS helps advance research in childhood epilepsies. We hope that the utilization of outcomes suggested by this COS as a framework in future studies will reduce reporting bias and allow for evidence to be synthesized across different studies.

## DISCLOSURE

None of the authors has any conflict of interest to disclose. We confirm that we have read the Journal's position on issues involved in ethical publication and affirm that this report is consistent with those guidelines.
