# Supplementary material for: Core Health Outcomes in Childhood Epilepsy (CHOICE): Development of a core outcome set using systematic review methods and a Delphi survey consensus
Source: Epilepsia. 2019 Apr 25;60(5):857–71. doi: 10.1111/epi.14735 (PMC6563436; doi:10.1111/epi.14735)
Supplement: Supplementary file 1 [file EPI-60-857-s001.pdf]

SEARCH LOG 1

DATABASE	DATE	Number of hits
CDSR	28/9/17	56
DARE	28/9/17	10
CENTRAL	28/9/17	60
MEDLINE	28/9/17	15
EMBASE	28/9/17	28
PSYCINFO	28/9/17	5
CINAHL	28/9/17	4

TOTAL: 147
DUPLICATES REMOVED: 33
NUMBER FOR REVIEW: 114 (RCTs 56; potential SRs 58)

Search strategies

Cochrane Library databases

CENTRAL

Search Name: Epilepsy outcomes
Date Run: 28/09/17 09:37:42.862
Description:

ID Search Hits
#1 MeSH descriptor: [Epilepsy, Rolandic] explode all trees 14
#2 rolandic:ti,ab 40
#3 BCECTS:ti,ab 2
#4 "centro temporal spikes":ti,ab 3
#5 "centrotemporal spikes":ti,ab 24
#6 (child* near/3 epilep*):ti,ab 611
#7 #1 or #2 or #3 or #4 or #5 62

CDSR/DARE

Search Name: Epilepsy outcomes
Date Run: 28/09/17 09:37:42.862
Description:

ID Search Hits
#1 MeSH descriptor: [Epilepsy, Rolandic] explode all trees 14
#2 rolandic:ti,ab 40
#3 BCECTS:ti,ab 2
#4 "centro temporal spikes":ti,ab 3
#5 "centrotemporal spikes":ti,ab 24
#6 (child* near/3 epilep*):ti,ab 611

#7 #1 or #2 or #3 or #4 or #5 62
#8 #1 or #2 or #3 or #4 or #5 or #6 643

MEDLINE
Database: Ovid MEDLINE(R) Epub Ahead of Print, In-Process & Other Non-Indexed Citations, Ovid MEDLINE(R) Daily and Ovid MEDLINE(R) <1946 to Present>
Search Strategy:
----
1 Epilepsy, Rolandic/ (543)
2 rolandic.ti,ab. (1530)
3 BCECTS.ti,ab. (53)
4 centro temporal spikes.ti,ab. (119)
5 centrotemporal spikes.ti,ab. (384)
6 1 or 2 or 3 or 4 or 5 (1952)
7 systematic.ti. (99433)
8 (systematic* adj2 review*).ab. (92392)
9 systematic overview.ti,ab. (845)
10 evidence synthesis.ti,ab. (2775)
11 (medline or pubmed).ab. (147225)
12 7 or 8 or 9 or 10 or 11 (229623)
13 6 and 12 (15)

EMBASE

Database: Embase <1974 to 2017 September 27>
Search Strategy:
----
1 rolandic epilepsy/ (726)
2 rolandic.ti,ab. (2129)
3 BCECTS.ti,ab. (89)
4 centro temporal spikes.ti,ab. (194)
5 centrotemporal spikes.ti,ab. (552)
6 1 or 2 or 3 or 4 or 5 (2835)
7 "systematic review (topic)"/ (22189)
8 systematic.ti. (114424)
9 (systematic* adj2 review*).ab. (113048)
10 systematic overview.ti,ab. (887)
11 evidence synthesis.ti,ab. (3064)
12 (medline or pubmed).ab. (176528)
13 7 or 8 or 9 or 10 or 11 or 12 (286135)
14 6 and 13 (28)

PsycINFO

Database: PsycINFO <1806 to September Week 4 2017>
Search Strategy:
----
1 rolandic.ti,ab. (492)
2 BCECTS.ti,ab. (29)
3 centro temporal spikes.ti,ab. (42)

4 centrotemporal spikes.ti,ab. (136)
5 1 or 2 or 3 or 4 (616)
6 systematic.ti. (18146)
7 (systematic* adj2 review*).ab. (18292)
8 systematic overview.ti,ab. (221)
9 evidence synthesis.ti,ab. (424)
10 (medline or pubmed).ab. (16611)
11 6 or 7 or 8 or 9 or 10 (36715)
12 5 and 11 (5)

CINAHL

Search ID#	Search Terms	Actions
S15	S6 AND S14	True S15 View Results (4) View Details Edit
S14	S7 OR S8 OR S9 OR S10 OR S11 OR S12 OR S13	True S14 View Results (101,022) View Details Edit
S13	AB medline or pubmed	True S13 View Results (43,843) View Details Edit
S12	TI (metasynthesis or meta-synthesis) OR AB (metasynthesis or meta-synthesis)	True S12 View Results (661) View Details Edit
S11	TI evidence synthesis OR AB evidence synthesis	True S11 View Results (1,611) View Details Edit
S10	TI (systematic N2 overview) OR AB (systematic N2 overview)	True S10 View Results (397) View Details Edit
S9	TI (systematic* N2 review*) OR AB (systematic* N2 review*)	True S9 View Results (60,487) View Details Edit
S8	TI systematic	True S8 View Results (44,192) View Details Edit
S7	(MH "Systematic Review")	True S7 View Results (42,512) View Details

		Edit
S6	S1 OR S2 OR S3 OR S4 OR S5	True S6 View Results (138)<br>View Details<br>Edit
S5	TI centrotemporal spikes OR AB centrotemporal spikes	True S5 View Results (41)<br>View Details<br>Edit
S4	TI "centro temporal spikes" OR AB "centro temporal spikes"	True S4 View Results (11)<br>View Details<br>Edit
S3	TI BCECTS OR AB BCECTS	True S3 View Results (5)<br>View Details<br>Edit
S2	TI rolandic OR AB rolandic	True S2 View Results (98)<br>View Details<br>Edit
S1	(MH "Epilepsy, Rolandic")	True S1 View Results (1)<br>View Details<br>Edit
