# Supplementary material for: Core Health Outcomes in Childhood Epilepsy (CHOICE): Development of a core outcome set using systematic review methods and a Delphi survey consensus
Source: Epilepsia. 2019 Apr 25;60(5):857–71. doi: 10.1111/epi.14735 (PMC6563436; doi:10.1111/epi.14735)
Supplement: Supplementary file 2 [file EPI-60-857-s002.pdf]

Table S2.1 - Characteristics of included papers

Study no.	Author (year) & (Reference no.)	Type of study/study design	Sample characteristics	Outcome domains	Outcome measures
**1**	Baca, C. B., et al. (2011).<br><br>(1)	**Observational**<br><br>Prospective, community-based cohort study of newly diagnosed childhood epilepsy, with ongoing prospective follow up.	• 277 children<br>129 females, 148 males.<br>• Mean age: 13 range: 8-17<br>• New diagnosed epilepsy, unprovoked seizure between 28 days and 15 years.	Health related quality of life<br><br>Cognitive function	Child health questionnaire (CHQ)<br><br>Neurologic medical records, parent interviews, school records, and standardized neuro-psychological testing using the Wechsler Intelligence Scale for Children.
**2**	Baca, C. B. et al., (2012)<br><br>(2)	**Observational**<br><br>Prospective community-based study of long-term outcomes of childhood-onset epilepsy. (The Connecticut Study of Epilepsy).	• 277 parent-child dyads<br>• Mean age of child: 5.1, SD: 2.5.<br>• Newly diagnosed childhood epilepsy.	Health related quality of life	Child health questionnaire (CHQ)
**3**	Bast et al., (2003)<br><br>(3)	**Systematic Review (SR)**<br><br>SR of the influence of sulthiame on EEG in BECTS	• 66 children<br>• Diagnosis of BECTS<br>• Mean age: 8.3, range 3-10 years	The rate of treatment failure events<br><br>EEG	Any first seizure<br><br>EEG
**4**	Benson, A., et al. (2016)<br><br>(4)	**Qualitative**<br><br>Mixed qualitative & quantitative in 2 stages.<br>Phase one: qualitative exploratory design using semi-structured interviews.<br><br>Phase 2: Questionnaires/cross-sectional survey	Phase one:<br>• 33 Children aged 6-16 years, mean age: 7.33 and 40 parents.<br>• Prescription of anti-epileptic medication<br><br>Phase two:<br>• 47 children aged 8-18 years; mean age 13.19, and 72 parents.	Themes: Social exclusion in epilepsy, Activity restrictions, Teasing/bullying, concealment, stigma coaching<br><br>Stigma	Interview<br><br>Child Stigma Scale (CSS)
**5**	Borggraefe et al., (2013)	**Randomised Controlled Trial (RCT)**	• 44 children between 6-12 years of age<br>• Diagnosis of BECTS	Primary endpoint: occurrence of a treatment failure event	Occurrence of a seizure during the observation period – 24 weeks after reaching target dose.

Study no.	Author (year) & (Reference no.)	Type of study/study design	Sample characteristics	Outcome domains	Outcome measures
	(5)	Randomised, double-blinded controlled trial (over 26 months). Non inferiority trial of LEV (Levetiricetam) compared to STM (Sulthiame). Randomly allocated to the LEV or STM treatment arm.	• LEV group (n=21): mean age: 8.7, 6 females<br><br>• STM group (n=22): mean age: 9.0, 10 females	Dropouts related to adverse events/occurrence of a drug related adverse reaction	Adverse events e.g. general symptoms, pain etc.<br>Urine analysis
**6**	Chong et al. (2016)<br><br>(6)	**SR of Qualitative Studies**<br><br>Systematic review of qualitative studies on the experiences of children and adolescence.	• 951 participants overall.<br>• Participants aged 3-21 years<br>• Diagnosed with epilepsy.	Themes: Loss of Bodily Control: Being overtaken, Susceptibility to physical harm, Fragility of the brain, Alertness to mortality, Bodily powerlessness, Loss of Privacy: Declarative Disease, Humiliating Involuntary Action, Unwanted special attention, Social embarrassment of medicine taking, Inescapable inferiority and discrimination:<br>Vulnerability to Prejudice, Consciousness of abnormality, Limiting social freedom, Therapeutic Burden and Futility: Awaiting a Fabled Remission, Overwhelming Life Disruption, Social and spiritual connectedness	Interview
**7**	Connock et al., (2006).<br><br>(7)	**SR**<br><br>SR to examine the clinical effectiveness and cost-effectiveness of newer anti-epileptic drugs (AEDs) for epilepsy in children. Drugs included: gabapentin,	• Young people with epilepsy under 18 years old and mixed age groups with epilepsy if including persons less than 18 years old.	Premature discontinuation due to adverse events<br><br>Seizure frequency	Adverse events e.g. vomiting, infection, headache, fever.<br><br>The number of seizures during maintenance

Supplementary 2. Charactersitics of included papers and provisional list of papers

Study no.	Author (year) & (Reference no.)	Type of study/study design	Sample characteristics	Outcome domains	Outcome measures
		lamotrigine, levetiricetam, oxcarbazepine, tiagabine, topiramate and vigabatrin.			
**8**	Coppola et al., (2007)<br><br>(8)	**RCT**<br><br>Randomised to levetiricetam (LEV) group, and an Oxcarbazepine (OXC) group.<br><br>LEV & OXC – 5mg/kg – up to a daily maximum dose of 20 mg/kg.	• 39 children aged between 3.3 to 14 years (m: 8.4) were randomised to receive monotherapy with LEV levetiricetam or OXC.<br>• New diagnosis of BECTS according to ILAEO, absence of neurological and mental deficits.	Complete seizure freedom<br><br>Awake & sleep EEG	Seizure freedom (recorded in an epilepsy diary)<br><br>EEG
**9**	Smith et al., (2015)<br><br>(9)	**Meta-Analysis**<br><br>Evaluation of 22 studies of literacy and/or language skills in children with rolandic epilepsy, published after 2000.	• Children and adolescents with rolandic epilepsy, aged 5-18 years.	Language & Literacy	Reading measured with: GORT accuracy (Gray, Oral, Reading Tests), WIAT (Weschsler Individual Achievement Test), WRAT (Wide Range Achievement Test)<br><br>Phonological processing measured with: TOWRE (rest of word reading efficacy)<br><br>Expressive language measured with: CELF (Clinical Evaluation of Language Fundamentals)<br><br>Receptive language measured with: CELF, Peabody Picture Vocabulary Test (PPVT)
**10**	Gutter et al., (2013)<br><br>(10)	**Observational**<br><br>Purpose of study were to explore the prevalence of	• 131 children – partial epilepsy aged 4 to 10 years and 161 age and sex matched controls.	Sleep	Sleep Disturbance Scale for children (SDSC), Medical Outcomes Study-Sleep Scale (MOSS –S) and Groningen

Supplementary 2. Charactersitics of included papers and provisional list of papers

Study no.	Author (year) & (Reference no.)	Type of study/study design	Sample characteristics	Outcome domains	Outcome measures
		sleep disturbances in a large cohort of school-age children with partial epilepsy.		Restrictions in childhood epilepsy<br><br>School environment/Peers & social support/autonomy & parent relations/psychological wellbeing/physical wellbeing	Sleep Quality Scale (GSQS)<br>Hague Seizure Severity Scale (HSSS), the Hague<br>Side Effects Scale (HaSES) and<br><br>Hague Restrictions in Childhood Epilepsy Scale (HaRCES)<br><br>Kidscreen-27
**11**	Kim et al., (2015)<br><br>(11)	**Observational**<br><br>Investigate cortical thickness and gray matter volume abnormalities in BECTS.	• 20 children (14 males, mean age: 7.5),<br>• Newly diagnosed BECTS	EEG<br><br>Cortical thickness/grey matter	EEG<br><br>MRI
**12**	Lewis et al., (2008)<br><br>(12)	**Observational**<br><br>E-survey and interviews	• 44 children and young people with epilepsy aged 3-23 (parent responses on behalf of child).<br>• Interviews with a separate group of 22 children and young people with epilepsy.	Impact of epilepsy on school	E-survey
**13**	Liu et al., (2016)<br><br>(13)	**RCT**<br><br>Children were randomised into two groups Group 1: topiramate once every night<br><br>Group 2: topiramate twice a day.	• 85 children<br>• 54 males and 31 females aged from 3.3 years to 13 years. Diagnosed with benign childhood epilepsy	EEG<br><br>Seizure frequency<br><br>Adverse events	EEG<br><br>Parent recorded<br><br>Parent recorded/physiological measures of blood, urine.
**14**	Loiselle et al., (2016)	**Observational**<br><br>Trajectories	• 120 children with epilepsy and their care giver	Paediatric epilepsy side-effects	Paediatric Epilepsy Side Effects Questionnaire (PESQ)<br>(Mortia et al., 2012).

Supplementary 2. Charactersitics of included papers and provisional list of papers

Study no.	Author (year) & (Reference no.)	Type of study/study design	Sample characteristics	Outcome domains	Outcome measures
	(14)	of health-related quality of life (HRQOL) among children with newly diagnosed epilepsy, and evaluate key predictors of HRQOL trajectories.	• 2-12 years old, received an epilepsy diagnosis on day of study recruitment	Health-related quality of life<br><br>Parental fears about the impact of a child's seizures on functioning	Care-giver proxy report version of the PedsQL 4.0 (Varni, Seid & Kurtin, 2001).<br><br>The Parent Report of Psychosocial Care (Austin, Dunn, Huster & Rose, 1998) – Includes the five-item Concerns and Fears subscale (assesses parental fears about the impact of the child's seizures on functioning and outcomes)
**15**	Melchionda et al., (1999)<br><br>(15)	**Observational**<br><br>Evaluate long term evolution of headaches associated with rolandic centrotemporal spikes	• 32 children with rolandic centro temporal spikes	Headache	Occurrence of symptom
**16**	Mitsudome et al., (1997<br><br>(16)	**Observational**<br><br>Effectiveness of clonazepam on rolandic discharges.	• 32 children with centro temporal spikes (CTS) that has episodes of headache (16 females) with centro-temporal spikes (CTS) at EEF<br>• 52 matched controls	EEG	EEG
**17**	Moffat et al., (2009)<br><br>(17)	**Observational**<br><br>Impact of childhood epilepsy on quality of life	• 22 children (11 male, 11 female)<br>• Mean age; 9, Range: 7-12<br>• Experiences partial seizures and some generalised seizures.	Themes: Impact on social life, developmental/role related issues, psychological epilepsy related issues, peer acceptance, school-related issues, academic impact, family reactions, adjustment, compliance, seizure experience, fear, coping, the future,	Interview

Supplementary 2. Charactersitics of included papers and provisional list of papers

Study no.	Author (year) & (Reference no.)	Type of study/study design	Sample characteristics	Outcome domains	Outcome measures
				translational periods, medical experiences, emotional impact	
**18**	Nevitt et al., (2016)<br><br>(18)	**SR**<br><br>RCT's with a comparison of carbamazepine monotherapy with phenobarbitone monotherapy in individuals with epilepsy	• Children or adults with partial onset seizures<br>• Individuals with a new diagnosis of epilepsy	Seizures<br><br>Adverse events<br><br>IQ<br><br>Memory	Incidence of seizures<br><br>Side effects, withdrawals, incidence of allergic reactions<br><br>WISC-R Scale, Bender-Gestalt test
**19**	Rating et al., (2000)<br><br>(19)	**RCT**<br><br>Children were randomized to receive either STM or a placebo	• 66 children<br>• Aged between 3-10 years<br>• Diagnosis of BECTS (or has 2 ore	Adverse events	Adverse event
**20**	Reilly et al., (2015)<br><br>(20)	**Observational**<br><br>Factors associated with quality of life in childhood epilepsy.	• Children recruited from the CHESS study (identification of children with 'active' epilepsy born between 1995 and 2007'.<br>• Children aged 5-15 years	Quality of life	QOLCE US
**21**	Ronen et al., (1999)<br><br>(21)	**Observational/qualitative**<br><br>Identifying the attributes of Health related quality of life (HRQOL) in childhood epilepsy.	• 29 children and their parents<br>• Mean age: 9.2<br>• Simple/complex partial seizures	Themes: Experience of epilepsy, life fulfilment/time use, home activities, school issues, and activities, social experiences, impact of epilepsy	Interview
**22**	Sajobi et al., (2017)<br><br>(22)	**Observational**<br><br>Trajectories across health related quality of life domains in children with new onset epilepsy	• 373 children with new-onset epilepsy	Family life event changes<br><br>Family functioning/resources	The Family Inventory of Life Event Changes (FILE)<br><br>Family inventory of resources and management (FIRM)

Supplementary 2. Charactersitics of included papers and provisional list of papers

Study no.	Author (year) & (Reference no.)	Type of study/study design	Sample characteristics	Outcome domains	Outcome measures
				Health-related quality of life<br><br>Epilepsy severity	QOLCE-55<br><br>Global Assessment of Severity of Epilepsy (GASE)<br><br>The Family adaptability, partnership, growth, affection and resolve (APGAR)
**23**	Schmitt et al., (2009)<br><br>(23)	**Observational**<br><br>Effects of valproic acid on sleep in children with epilepsy	• 46 children<br>• Aged 1.7-17.4 years, mean 8.4<br>• History of generalised epilepsies	Sleep	Sleep habits survey
**24**	Schraegle et al., (2016)<br><br>(24)	**Observational**<br><br>Executive functioning and HRQOL in childhood epilepsy	• 130 children<br>• Mean age: 11.6 years, SD: 3.6 years<br>• Epilepsy diagnosis	Executive functioning<br><br>Behaviour<br><br>Memory<br><br>Intelligence<br><br>HRQOL	Behaviour rating inventory of executive functioning (BRIEF)<br><br>BRIEF<br><br>BRIEF<br><br>Wechsler Intelligence Scales (WSI-II) (WISC-IV)<br><br>Quality Of Life in Childhood Epilepsy scale (QOLCE)
**25**	Seidel et al., (1999)<br><br>(25)	**Observational**<br><br>Cognitive and behavioural effects of carbamazepine in rolandic children.	• 27 Children<br>• 12 diagnosed with benign rolandic epilepsy or migraine headache, 15 with migraine<br>• Mean age BECTS: 9.7, aged between 6-12 years	Intelligence<br><br>Language<br><br>Memory<br><br>Motor skills	Wechsler Intelligence Scale for Children 3rd edition<br><br>Boston Naming Test<br><br>Wide Range Assessment of Memory and Learning<br><br>Finger tapping test, Grooved pegboard test

Supplementary 2. Charactersitics of included papers and provisional list of papers

Study no.	Author (year) & (Reference no.)	Type of study/study design	Sample characteristics	Outcome domains	Outcome measures
				Behaviour	Child behaviour checklist (CBCL)
**26**	Stafstrom et al., (2012)<br><br>(26)	**Observational**<br><br>Art therapy focus groups for young people with epilepsy	• 16 children, mean age: 12.8 years, SD: 2.9<br>• Range of childhood epilepsies including rolandic	Self-concept<br><br><br><br><br><br><br><br>Attitude towards epilepsy<br><br><br><br><br>Impact of epilepsy	Piers-Harris Children's Self-Concept Scale 2<br><br>Art therapy assessments (Seizure drawing task, Formal Elements Art Therapy Scale/Picking an Apple from a Tree, Levick Emotional and Cognitive Art Therapy Assessment)<br>Child Attitude Towards Illness Scale (CATIS)<br><br><br><br>Impact of Childhood Neurologic disability scale (ICNDS)/ Impact of Paediatric Epilepsy Scale (IPES)
**27**	Tacke et al., (2016)<br><br>(27)	**RCT**<br><br>Children with BECTS Randomly allocated to LEV or STM treatment group.	• 44 children<br>• Aged between 6 and 12 years of age<br>• Diagnosis of BECTS	Memory	Number recall test from the Kaufman assessment battery for children (K-ABC), Verbal learning memory test (CLMT), culture free intelligence test (CFT-R)
**28**	Tan et al., (2014)<br><br>(28)	**SR**<br><br>Review of antiepileptic drug RCT in children with BECTS	• 262 children up to the age of 15 years<br>• Diagnosis of BECTS	Proportion of patients seizure free at 3 & 12 months<br><br><br>Adverse event	Proportion of patients seizure free at 3 & 12 months<br><br><br>Adverse event
**29**	Tedrus et al., (2006)<br><br>(29)	**Observational**<br><br>EEG and intelligence in children with BECTS	• 26 children aged 8-11 years old (10 female, 16 male) & a paired control group<br>• Diagnosis of BECTS	Intelligence<br><br><br><br><br>EEG	Wechsler Intelligence Scale for Children (WISC-III)<br><br><br><br>EEG

Supplementary 2. Charactersitics of included papers and provisional list of papers

Study no.	Author (year) & (Reference no.)	Type of study/study design	Sample characteristics	Outcome domains	Outcome measures
**30**	Turky et al., (2008)<br><br>(30)	**Observational**<br><br>Epidemiological study aimed at determining the prevalence of behavioural and emotional problems in a UK community-based population of children and adolescents with epilepsy	• 56 children and adolescents with epilepsy aged 4-17.<br>• 25 males, 31 females.<br>• Diagnosis of epilepsy, age at epilepsy onset: 6 years old.	Strengths & difficulties<br><br>Seizure severity<br><br>Moods, feelings & emotions<br><br>Impact of paediatric epilepsy<br><br>Quality of life in epilepsy	Strengths & difficulties questionnaire (SDQ)<br><br>Revised Liverpool Seizure Severity Scale<br><br>The Moods and Feelings Questionnaire (MFQ)<br><br>The Impact of Paediatric epilepsy scale (IPES)<br><br>Quality of Life in Epilepsy Inventory for Adolescents (QOLIE-AD-48)
**31**	Turner et al., (2004)<br><br>(31)	**Observational**<br><br>Prospective, randomized, single-blinded, crossover, placebo-controlled, pilot clinical trial investigating exposure to music on EEG of children with BECTS.	• 4 children aged 5-9 years, were selected from EEGs diagnostic of BECTS	Interictal epileptiform discharges (IED)	EEG
**32**	Verhey et al., (2009)<br><br>(32)	**Observational**<br><br>QOL in children with epilepsy and agreement between child and parent	• Children aged 8-17 years were included with a diagnosis of active epilepsy<br>• 375 children and 378 parents	Health related quality of life	CHEQOL-25

Supplementary 2. Charactersitics of included papers and provisional list of papers

Study no.	Author (year) & (Reference no.)	Type of study/study design	Sample characteristics	Outcome domains	Outcome measures
**33**	Wille et al., (2010)<br><br>(33)	**Preference based/HRQOL measure**<br><br>Development of the EQ-5D-Y	• Epilepsy measure designed for young people	Mobility<br><br>Looking after myself<br><br>Usual activities<br><br>Pain/discomfort and feeling worried<br><br>Sad or unhappy	EQ-5DY
**34**	Stevens et al., (2011)<br><br>(34)	**Preference based/HRQOL measure**<br><br>Development of the Child Health Utility (CHU9D)	• 247 children recruited from general and clinical paediatric populations tested	Sad<br><br>Pain<br><br>Tired<br><br>Annoyed<br><br>School Work<br><br>Sleep<br><br>Daily routines and activities	CHU9D
**35**	Feeny et al., (2002)<br><br>(35)	**Preference based/HRQOL measure**<br><br>Development of the Health Utilities Index Mark 3 (HUI3)	• A random sample of the general population (≥16 years of age) in Hamilton, Ontario, Canada completed preference surveys	Vision<br><br>Hearing<br><br>Speech<br><br>Ambulation<br><br>Dexterity<br><br>Emotion	HUI3

Supplementary 2. Charactersitics of included papers and provisional list of papers

Study no.	Author (year) & (Reference no.)	Type of study/study design	Sample characteristics	Outcome domains	Outcome measures
				Cognition<br><br>Pain	
**36**	Mulhern et al., (2012)<br><br>(36)	**Preference based/HRQOL measure**<br><br>NEWQoL-6D	Epilepsy measure designed for adults<br>• Used sample of 1611 respondents with newly diagnosed epilepsy from SANAD study to generate classification system	Worry about attacks<br><br>Depression<br><br>Memory<br><br>Concentration<br><br>Stigma<br><br>Control	NewQol-6d
**37**	Sadeghi et al., 2014<br><br>(37)	**SR of outcome measures**	Conceptual analysis – no participants<br><br>Conceptual/theoretical analysis – no participants		DISABKIDS (Epilepsy Module)<br><br>ECQ (Epilepsy and children questionnaire)<br><br>ELQOL (epilepsy and learning disabilities quality of life)<br><br>EFA (Epilepsy Foundations of American Concerns Index)<br><br>Glasgow Epilepsy Outcome Scale (GEOS-C)<br><br>ICI (Impact of Childhood Illness)<br><br>ICNDS (Impact of Childhood Neurologic Disability)<br><br>IPES (Impact of Paediatric epilepsy scale)<br><br>QOLCE

Supplementary 2. Charactersitics of included papers and provisional list of papers

Study no.	Author (year) & (Reference no.)	Type of study/study design	Sample characteristics	Outcome domains	Outcome measures
					QOLIE-AD<br><br>QOLPES (Quality of life in paediatric epilepsy)<br><br>PedsQL<br><br>Neuro-QOL

Supplementary 2. Charactersitics of included papers and provisional list of papers

Table S2.2 - Provisional list of outcomes identified from systematic review and provided to the team

COMET Taxonomy	Outcome Domain	Aspects of health	Outcome identified verbatim in individual study	Study number of papers retrieved from systematic review (See Table 1)
**Physiological: Nervous System Outcomes**	Seizures	Seizure remission	Proportion seizure free	8, 18, 28
			Proportion of patients who are seizure free at 3 months, 12 months	28
			Complete seizure freedom	8
		Seizure frequency	Seizure frequency	7, 13, 18
			Seizure frequency and severity	11
			Mean seizure frequency per month	18, 20
		Seizure duration	Mean seizure duration	20, 18
		Seizure severity	Seizure severity	10, 22, 30, 37
			Severity of epilepsy	24, 22
		Seizure control	Seizure control	18

COMET Taxonomy	Outcome Domain	Aspects of health	Outcome identified verbatim in individual study	Study number of papers retrieved from systematic review (See Table 1)
**Physical Functioning**	Sleep	Sleep duration	Overall sleep	10
			Sleep start time & end	23
			Assumed sleep per night	23
			Actual sleep per night	23
			Assumed sleep per nap	23
			Actual sleep time per nap	23
			No. of hours of sleep	10
			Nap per day	23
			Sleep	34
		Sleep quality	Disorders of initiating and maintaining sleep	10
			Sleep breathing disorders	10
			Sleep quality	10
			Sleep hyperhidrosis	10
			Sleep disturbance/insomnia	10
			Disorders of arousal	10
			Sleep-wake transition disorders	10, 23
			Breathing problems/headache in sleep	10

Supplementary 2. Charactersitics of included papers and provisional list of papers

COMET Taxonomy	Outcome Domain	Aspects of health	Outcome identified verbatim in individual study	Study number of papers retrieved from systematic review (See Table 1)
		Sleep efficiency	Sleep adequacy	10
			Morningness/eveningness	23
		Daytime sleepiness	Sleepiness	23
			Disorders of excessive somnolence	10
			Daytime somnolence	10
			Energy/fatigue	20, 37
			Tired	34
	Physical health	Pain	Bodily pain/discomfort	1, 2
			Pain/discomfort – feeling worried	33
			Pain	34, 35, 37,
		Physical functioning	Physical functioning	1, 14, 22, 30, 37
			Physical restrictions	10, 20, 37
			Physical wellbeing	10
			Physical impact of epilepsy	21
			Role/social limitations physical	1, 2
				1, 2, 37
			Change in health	
				37

Supplementary 2. Charactersitics of included papers and provisional list of papers

COMET Taxonomy	Outcome Domain	Aspects of health	Outcome identified verbatim in individual study	Study number of papers retrieved from systematic review (See Table 1)
			Physical health	
	Manual ability	Manual ability	Mobility	33
			Ambulation	35
			Dexterity	35
			Motor skills	25
**Social Functioning**	Usual activities	Usual activities	Usual activities	33
		Recreation & Leisure	Activity restrictions	4
			Social activities	20, 37
		Self-care	Looking after self	33
			Independence	37
	Behaviour	Behaviour	Behaviour	1, 2, 30, 24
			Behavioural regulation	24
			Internalizing behaviour	25
			Externalizing behaviour	25
			Self-concept: behavioural adjustment	26
			Conduct problems	30

Supplementary 2. Charactersitics of included papers and provisional list of papers

COMET Taxonomy	Outcome Domain	Aspects of health	Outcome identified verbatim in individual study	Study number of papers retrieved from systematic review (See Table 1)
			Prosocial behaviour	30
			Peer problems	30
			Parental ratings of behaviour	25
			Social behaviour	30, 37
			Social health: sociability	37
			Social health: social role performance	37
			Role/social limitations: behavioural	1, 2
		Family functioning & cohesion	Family cohesion	1, 2
			Family activities	1, 2
			Family resources	22
			Parent impact – time/emotion	1, 2, 37
		Educational participation	School environment	10, 14
	Social	Social life/Engagement with friends & peers	Social exclusion in epilepsy	4
			Teasing/bullying	4, 21

Supplementary 2. Charactersitics of included papers and provisional list of papers

COMET Taxonomy	Outcome Domain	Aspects of health	Outcome identified verbatim in individual study	Study number of papers retrieved from systematic review (See Table 1)
			Social embarrassment of medicine taking	6
			Limiting social freedom	6
			Social life	17
			Social interactions	20, 37
			Peers & Social support	10
			Social functioning	14, 22, 30, 37
			Social interactions	20, 37
			Peer problems	30
			Social support	30, 37
			Interpersonal/social	32, 37
			Teasing/bullying	4, 21
			Communication	26, 30, 37
			Social impact of epilepsy/Impact on social activities	37
		Stigma	Social embarrassment of medicine taking	6
			Inescapable inferiority and discrimination: vulnerability to prejudice	6
			Unwanted special attention	6

Supplementary 2. Charactersitics of included papers and provisional list of papers

COMET Taxonomy	Outcome Domain	Aspects of health	Outcome identified verbatim in individual study	Study number of papers retrieved from systematic review (See Table 1)
			Stigma	4, 36, 37, 20, 30
**Role functioning**	Family relationships	Autonomy & relationships with parents	Family relationships & functioning	22, 37
			Autonomy & parent relations	10
			Family life event changes	22
			Autonomy	NF
			Family	NF
			Impact of epilepsy on relationships with family, peers and siblings	37
**Emotional functioning/wellbeing**	Mental Health	Behavioural difficulties	Behavioural difficulties	30
		Depression	Depression	20, 23, 36, 22, 34, 33
			Sad	34
			Sad or unhappy	33
		Anxiety	Anxiety	20, 37
		Self-esteem	Self-esteem	1, 2, 20, 37
			Over-all self-concept	26
			Self-concept: physical appearance and attributes	26

Supplementary 2. Charactersitics of included papers and provisional list of papers

COMET Taxonomy	Outcome Domain	Aspects of health	Outcome identified verbatim in individual study	Study number of papers retrieved from systematic review (See Table 1)
		Mental wellbeing	Mental health	1, 2
			Psychological wellbeing	10
			Emotional control	24
			Self-concept: happiness & satisfaction	26
			Moods, feelings and emotions (over 2 weeks)	30, 37
			Mood/Mood state	37
			Intrapersonal/emotional	32
			Annoyed	34, 37
			Emotion	35
			Optimism	37
			Emotional functioning	37
			Attitude towards epilepsy	26, 30, 37
			Fear of seizures	NF
			Psychological functioning	14
			Control/helplessness	20, 37
			Impact of epilepsy (psychosocial)	30, 26
			Emotional disorders	30

Supplementary 2. Charactersitics of included papers and provisional list of papers

COMET Taxonomy	Outcome Domain	Aspects of health	Outcome identified verbatim in individual study	Study number of papers retrieved from systematic review (See Table 1)
			Role/social limitations emotional	1, 2
			Epilepsy impact: effects of epilepsy and antiepileptic medications	30
			Disclosure & normality	6, 32, 37
		Secrecy	Secrecy	32, 4
			Concealment	4
		Fears about epilepsy	Parental fears about the impact of a child's seizure	14
			Worries/concerns	32, 34, 36, 37
			Concerns about epilepsy	37
			Future worries	37
			Fear of seizures	37
			Parental concern	37
			Impact of seizures	37
			Prospects for the future	37
**Cognitive Functioning**	Cognition	Language & Literacy	Reading	9
			Phonological processing	9

Supplementary 2. Charactersitics of included papers and provisional list of papers

COMET Taxonomy	Outcome Domain	Aspects of health	Outcome identified verbatim in individual study	Study number of papers retrieved from systematic review (See Table 1)
			Expressive language	9, 20, 25, 37
			Receptive language	9, 20, 25, 37
			Speech	35
		Memory	Memory	18, 22, 30, 25, 27, 30, 36, 37
			Working memory	24
		Intelligence	IQ scores	18, 24, 25, 29
		Concentration	Attention/concentration	20, 30, 36, 25, 37
			Hyperactivity/inattention	30
		Executive functioning	Metacognitive index	24
			Planning/organisation	24
			Executive functioning (Global executive composite)	24
		Cognition	Speeded visual search and mental flexibility	25
			Cognition	35
			Cognitive function	1, 37, 22
			Control	36

Supplementary 2. Charactersitics of included papers and provisional list of papers

COMET Taxonomy	Outcome Domain	Aspects of health	Outcome identified verbatim in individual study	Study number of papers retrieved from systematic review (See Table 1)
			Cognitive effects of epilepsy	37
			Applied cognition: general concerns	37
			Speeded visual search and mental flexibility	25
			Other cognitive	37
		School performance	Self-concept: Intellectual and school status	26
			School/scholastic functioning	37
			Education	37
			Impact of epilepsy on academic achievement	37
			Impact of epilepsy on school	10
			Academic self-concept:	26
**Global quality of life**	Global quality of life	Global quality of life	Global general health	1, 2, 14, 20, 22, 30, 32, 37
			General health perceptions	1, 2, 30, 37
			Impact of epilepsy on health	37
			General/overall health	37
			Overall impact of epilepsy	37

Supplementary 2. Charactersitics of included papers and provisional list of papers

COMET Taxonomy	Outcome Domain	Aspects of health	Outcome identified verbatim in individual study	Study number of papers retrieved from systematic review (See Table 1)
			Total impact of childhood neurologic disability	37
			Health related quality of life	14, 20, 30, 32, 37
**Adverse events/effects**	Adverse events	Unintended adverse events	Routine urine test	13, 5
			Liver and renal function	13
			Weight	13
			Routine blood test	13
			Paediatric epilepsy side effects	14, 18, 10
			Adverse events	18, 19, 28, NF, 5, 7, 13, 15, 30, 35, 37, 11
			Anti-epileptic drug side effects	37, 10
			Seizure effects	37
			Headache	15
			Hearing	35
			Vision	35
			Safety/injury	37

Supplementary 2. Charactersitics of included papers and provisional list of papers

References

1. Baca CB, Vickrey BG, Caplan R, Vassar SD, Berg AT. Psychiatric and medical comorbidity and quality of life outcomes in childhood-onset epilepsy. Pediatrics. 2011;peds-2011.
2. Baca CB, Vickrey BG, Vassar SD, Berg AT. Seizure recency and quality of life in adolescents with childhood-onset epilepsy. Epilepsy Behav. 2012;23(1):47–51.
3. Bast T, Völp A, Wolf C, For The Sulthiame Study Group DR. The influence of sulthiame on EEG in children with benign childhood epilepsy with centrotemporal spikes (BECTS). Epilepsia. 2003;44(2):215–20.
4. Benson A, O'Toole S, Lambert V, Gallagher P, Shahwan A, Austin JK. The stigma experiences and perceptions of families living with epilepsy: implications for epilepsy-related communication within and external to the family unit. Patient Educ Couns. 2016;99(9):1473–81.
5. Borggraefe I, Bonfert M, Bast T, Neubauer BA, Schotten KJ, Maßmann K, et al. Levetiracetam vs. sulthiame in benign epilepsy with centrotemporal spikes in childhood: a double-blinded, randomized, controlled trial (German HEAD Study). Eur J Paediatr Neurol. 2013;17(5):507–14.
6. Chong L, Jamieson NJ, Gill D, Singh-Grewal D, Craig JC, Ju A, et al. Children's experiences of epilepsy: a systematic review of qualitative studies. Pediatrics. 2016;138(3):e20160658.
7. Cummins C. The clinical effectiveness and cost-effectiveness of newer drugs for children with epilepsy. Inf Sci. 121(414):6769.
8. Coppola G, Franzoni E, Verrotti A, Garone C, Sarajlija J, Operto FF, et al. Levetiracetam or oxcarbazepine as monotherapy in newly diagnosed benign epilepsy of childhood with centrotemporal spikes (BECTS): an open-label, parallel group trial. Brain Dev. 2007;29(5):281–4.
9. Smith AB, Bajomo O, Pal DK. A meta-analysis of literacy and language in children with rolandic epilepsy. Dev Med Child Neurol. 2015;57(11):1019–26.
10. Gutter T, Brouwer OF, de Weerd AW. Subjective sleep disturbances in children with partial epilepsy and their effects on quality of life. Epilepsy Behav. 2013;28(3):481–8.
11. Kim E-H, Yum M-S, Shim W-H, Yoon H-K, Lee Y-J, Ko T-S. Structural abnormalities in benign childhood epilepsy with centrotemporal spikes (BCECTS). Seizure. 2015;27:40–6.
12. Lewis A, Parsons S. Understanding of epilepsy by children and young people with epilepsy. Eur J Spec Needs Educ. 2008;23(4):321–35.
13. Liu C, Song M, Wang J. Nightly oral administration of topiramate for benign childhood epilepsy with centrotemporal spikes. Child's Nerv Syst. 2016;32(5):839–43.
14. Loiselle KA, Ramsey RR, Rausch JR, Modi AC. Trajectories of health-related quality of life among children with newly diagnosed epilepsy.

Supplementary 2. Charactersitics of included papers and provisional list of papers

J Pediatr Psychol. 2016;41(9):1011–21.
15. Melchionda D, Verrotti A, Chiarelli F, Domizio S, Sabatino G, Mucedola T, et al. Headache in children with centrotemporal spikes. Neurophysiol Clin Neurophysiol. 1999;29(1):90–100.
16. Mitsudome A, Ohfu M, Yasumoto S, Ogawa A, Hirosea S, Ogata H, et al. The effectiveness of clonazepam on the Rolandic discharges. Brain Dev. 1997;19(4):274–8.
17. Moffat C, Dorris L, Connor L, Espie CA. The impact of childhood epilepsy on quality of life: A qualitative investigation using focus group methods to obtain children's perspectives on living with epilepsy. Epilepsy Behav. 2009;14(1):179–89.
18. Nevitt SJ, Marson AG, Weston J, Tudur Smith C. Carbamazepine versus phenobarbitone monotherapy for epilepsy: an individual participant data review. Cochrane Libr. 2016;
19. Wolf C, Bast T. Sulthiame as monotherapy in children with benign childhood epilepsy with centrotemporal spikes: a 6-month randomized, double-blind, placebo-controlled study. Epilepsia. 2000;41(10):1284–8.
20. Reilly C, Atkinson P, Das KB, Chin RFM, Aylett SE, Burch V, et al. Factors associated with quality of life in active childhood epilepsy: a population-based study. Eur J Paediatr Neurol. 2015;19(3):308–13.
21. Ronen GM, Rosenbaum P, Law M, Streiner DL. Health-related quality of life in childhood epilepsy: the results of children's participation in identifying the components. Dev Med Child Neurol. 1999;41(8):554–9.
22. Sajobi TT, Wang M, Ferro MA, Brobbey A, Goodwin S, Speechley KN, et al. Multivariate trajectories across multiple domains of health-related quality of life in children with new-onset epilepsy. Epilepsy Behav. 2017;75:72–8.
23. Schmitt B, Martin F, Critelli H, Molinari L, Jenni OG. Effects of valproic acid on sleep in children with epilepsy. Epilepsia. 2009;50(8):1860–7.
24. Schraegle WA, Titus JB. Executive function and health-related quality of life in pediatric epilepsy. Epilepsy Behav. 2016;62:20–6.
25. Seidel WT, Mitchell WG. Cognitive and behavioral effects of carbamazepine in children: data from benign rolandic epilepsy. J Child Neurol. 1999;14(11):716–23.
26. Stafstrom CE, Havlena J, Krezinski AJ. Art therapy focus groups for children and adolescents with epilepsy. Epilepsy Behav [Internet]. 2012;24(2):227–33. Available from: http://dx.doi.org/10.1016/j.yebeh.2012.03.030
27. Tacke M, Gerstl L, Heinen F, Heukaeufer I, Bonfert M, Bast T, et al. Effect of anticonvulsive treatment on neuropsychological performance in children with BECTS. Eur J Paediatr Neurol. 2016;20(6):874–9.
28. Tan HJ, Singh J, Gupta R, de Goede C. Comparison of antiepileptic drugs, no treatment, or placebo for children with benign epilepsy with centro temporal spikes. Cochrane Database Syst Rev. 2014;9(9):Cd006779.
29. Tedrus GMAS, Fonseca LC, Tonelotto JMF, Costa RM, Chiodi MG. Benign Childhood Epilepsy with Centra-Temporal Spikes: Quantitative EEG and the Wechsler Intelligence Scale for Children (WISC-III). Clin EEG Neurosci. 2006;37(3):193–7.

Supplementary 2. Charactersitics of included papers and provisional list of papers

30. Turky A, Beavis JM, Thapar AK, Kerr MP. Psychopathology in children and adolescents with epilepsy: an investigation of predictive variables. Epilepsy Behav. 2008;12(1):136–44.
31. Turner RP. The acute effect of music on interictal epileptiform discharges. Epilepsy Behav. 2004;5(5):662–8.
32. Verhey LH, Kulik DM, Ronen GM, Rosenbaum P, Lach L, Streiner DL. Quality of life in childhood epilepsy: what is the level of agreement between youth and their parents? Epilepsy Behav. 2009;14(2):407–10.
33. Wille N, Badia X, Bonsel G, Burström K, Cavrini G, Devlin N, et al. Development of the EQ-5D-Y: a child-friendly version of the EQ-5D. Qual life Res. 2010;19(6):875–86.
34. Stevens K. Assessing the performance of a new generic measure of health-related quality of life for children and refining it for use in health state valuation. Appl Health Econ Health Policy. 2011;9(3):157–69.
35. Feeny D, Furlong W, Torrance GW, Goldsmith CH, Zhu Z, DePauw S, et al. Multiattribute and single-attribute utility functions for the health utilities index mark 3 system. Med Care. 2002;40(2):113–28.
36. Mulhern B, Rowen D, Jacoby A, Marson T, Snape D, Hughes D, et al. The development of a QALY measure for epilepsy: NEWQOL-6D. Epilepsy Behav. 2012;24(1):36–43.
37. Sadeghi S, Fayed N, Ronen GM. Patient-reported outcome measures in pediatric epilepsy: A content analysis using World Health Organization definitions. Epilepsia. 2014;55(9):1431–7.

Supplementary 2. Charactersitics of included papers and provisional list of papers
