# Supplementary material for: Core Health Outcomes in Childhood Epilepsy (CHOICE): Development of a core outcome set using systematic review methods and a Delphi survey consensus
Source: Epilepsia. 2019 Apr 25;60(5):857–71. doi: 10.1111/epi.14735 (PMC6563436; doi:10.1111/epi.14735)
Supplement: Supplementary file 3 [file EPI-60-857-s003.pdf]

S3.1 - Demographics of participants from R2 of the Delphi survey

Characteristic	N (%)
**Professionals**	61 (100)
Place of residence in UK	
North East	3 (5)
North West	1 (2)
Yorkshire and The Humber	4 (7)
East Midlands	2 (3)
West Midlands	3 (5)
East of England	2 (3)
London	18 (30)
South East	5 (8)
South West	9 (15)
Scotland	3 (5)
Wales	5 (8)
Ireland	4 (7)
Outside UK	2 (3)
Ethnicity	
White	44 (72)
Black	2 (3)
Asian	11 (18)
Hispanic/Latino	1 (2)
Mixed race	1 (2)
Other	2(3)
**Parents & Young people**	19 (100)
Place of residence in UK	
North East	0 (0)
North West	0 (0)
Yorkshire and The Humber	1 (5)
East Midlands	1 (5)
West Midlands	0 (0)
East of England	0 (0)
London	4 (21)
South East	2 (11)
South West	3 (16)
Scotland	0 (0)
Wales	0 (0)
Ireland	4 (21)
Outside UK	4 (21)
Ethnicity	
White	16 (84)
Black	2 (11)

Characteristic	N (%)
Asian	0 (0)
Hispanic/Latino	0 (0)
Mixed race	0 (0)
Other	1 (5)
Average child age	10.5, (s.d. 1.9)
