# Supplementary material for: Core Health Outcomes in Childhood Epilepsy (CHOICE): Development of a core outcome set using systematic review methods and a Delphi survey consensus
Source: Epilepsia. 2019 Apr 25;60(5):857–71. doi: 10.1111/epi.14735 (PMC6563436; doi:10.1111/epi.14735)
Supplement: Supplementary file 4 [file EPI-60-857-s004.pdf]

Table S4.1 – Outcomes suggested after R1 of the Delphi survey

Additional outcome suggested	Stakeholder & Score	Score	Notes
**Frequency of hospital out-patient attendance for BECTS review**	Professional	7	New outcome identified<br><br>**Outcome:** Attendance for medical appointments in outpatients<br>**Domain:** Seizures<br>**Description:** Routine attendances for medical epilepsy management
**seen by consultant with specialist expertise in epilepsy**	Professional	7	No new outcomes represented in free-text response/already covered in outcomes.
**seen by consultant paediatric neurologist**	Professional	7	No new outcomes represented in free-text response/already covered in outcomes.
**seen by paediatric epilepsy specialist nurse**	Professional	7	No new outcomes represented in free-text response/already covered in outcomes.
**taking anti-convulsant for BECTS**	Professional	8	No new outcomes represented in free-text response/already covered in outcomes.
**Hospital attendances**	Professional	4	New outcome identified<br><br>**Outcome:** Unplanned hospital attendances at Accident & Emergency<br>**Domain:** Adverse events<br>**Description:** Visiting the hospital

Additional outcome suggested	Stakeholder & Score	Score	Notes
			due to an acute medical emergency
**Hospital admissions**	Professional	4	New outcome identified<br>**Outcome:** Unplanned epilepsy-related admission to hospital as inpatient<br>**Domain:** Adverse Events<br>**Description**: Unexpectedly needing to be admitted to hospital
**Change in medication; e.g. withdrawal or addition of AED**	Professional	5	New outcome identified<br>**Outcome:** Drug treatment failure (adverse events or poor seizure control)<br>**Domain:** Adverse Events<br>**Description**: stopping medication because it's not working or causing problems
**Epilepsy in this person drug resistant (by ILAE criteria)**	Professional	4	No new outcomes represented in free-text response/already covered in outcomes.
**list comorbidities**	Professional	7	No new outcomes represented in free-text response/already covered in outcomes.
**Evidence of parasomnia with awakening at night**	Professional	7	No new outcomes represented in free-text response/already covered in outcomes.
**Percentage of diagnosed**	Professional	9	No new outcomes represented in

Additional outcome suggested	Stakeholder & Score	Score	Notes
**comorbidities in children with RE**			free-text response/already covered in outcomes.
**The types of seizures seen in the child with the diagnosis**	Professional	6	No new outcomes represented in free-text response/already covered in outcomes.
**The percentage of children with RE whose epilepsy evolves or becomes uncontrollable.**	Professional	9	No new outcomes represented in free-text response/already covered in outcomes.
**speech regression / aphasia**	Professional	9	No new outcomes represented in free-text response/already covered in outcomes.
**Awareness of SUDEP**	Parent	8	No new outcomes represented in free-text response/already covered in outcomes.
**TICS**	Parent	8	No new outcomes represented in free-text response/already covered in outcomes.
**Improvement of deterioration of school work after starting treatment**	Professional	6	No new outcomes represented in free-text response/already covered in outcomes.
**Ability to play Sport**	Parent	4	No new outcomes represented in free-text response/already covered in outcomes.
