# Supplementary material for: Core Health Outcomes in Childhood Epilepsy (CHOICE): Development of a core outcome set using systematic review methods and a Delphi survey consensus
Source: Epilepsia. 2019 Apr 25;60(5):857–71. doi: 10.1111/epi.14735 (PMC6563436; doi:10.1111/epi.14735)
Supplement: Supplementary file 5 [file EPI-60-857-s005.pdf]

Supplementary 5 – Consensus meeting minutes and participants

Table S5.1 – Participants present at the consensus meeting

Initials	Meeting role	Stakeholder Group	Membership
CM	**Meeting facilitator/chair**	**n/a**	**Research team**
HC	**Assistant Facilitator**	**n/a**	**Research team**
SI	**Assistant**	**n/a**	**King's College London**
SL	**Family Engagement Officer**	**n/a**	**Research team**
DKP	**Participant**	**Professional (Paediatric neurologist)**	**Delphi survey participant/ Research team**
PG	**Participant**	**Professional (consultant in sleep medicine)**	**Delphi survey participant/ Research team**
BC	**Participant**	**Professor of children's nursing**	**Research team/voted in meeting**
JH	**Participant**	**Professional (Paediatrician)**	**Delphi survey participant**
DJ	**Participant**	**Professional (Consultant in sleep medicine)**	**Delphi survey participant**
CT	**Participant**	**Professional (Clinical Psychologist)**	**Delphi survey participant (R1)**
SM	**Participant**	**Professional (Physiologist)**	**Delphi survey participant**
RW	**Participant**	**Professional (Paediatric neurologist)**	**Delphi survey participant**
DG	**Participant**	**Professional (Paediatrician)**	**Delphi survey participant**
MS	**Participant**	**Parent of AS**	**Delphi survey participant**
AS	**Participant**	**Young Person**	**Delphi survey participant**
CT	**Participant**	**Parent of BT**	**Delphi survey participant**
BT	**Participant**	**Young Person**	**Parent was a Delphi survey participant**
DR	**Participant**	**Parent**	**Research team**
JC	**Participant**	**Parent**	**Research team**

S5.2 Minutes of consensus meeting

CHOICE – Core Health Outcomes in Childhood Epilepsy Consensus Meeting

Facilitators:
1. Christopher Morris
2. Holly Crudgington

Assistants:
1. SL (Family Engagement Officer)
2. SI (Assistant)

Eligible to vote:
Professionals (n=9)

1. RW (Paediatric neurologist)
2. DJ (Consultant in Sleep medicine)
3. BC (Professor of Children's Nursing)
4. CT (Clinical Psychologist)
5. PG (Consultant in Sleep medicine)
6. DP (Paediatric neurologist)
7. JH (Paediatrician)
8. DG (Paediatrician)
9. SM (Physiologist)

Parents (n=4)

1. JC
2. DR
3. CT
4. MS

Young People (n=2)

1. AS
2. BT

Total present at meeting: 19
Total voting: 15

Outcomes in from the Delphi, prior to meeting (n=11)

Outcome 1. Seizure Freedom
Outcome 2. Seizure Frequency
Outcome 3. Seizure duration
Outcome 4. Memory
Outcome 5. Self-harm
Outcome 6. Fears of having a seizure
Outcome 7. Learning
Outcome 8. Concentration
Outcome 9. Overall Quality of Life
Outcome 10. Adverse events or reactions
Outcome 11. Drug treatment failure events (adverse events or poor seizure control)

Outcomes voted in after the meeting (n= 28)

Outcome 12. Seizure Severity
Outcome 5. Total time spent asleep at night
Outcome 6. Total time spent asleep in 24 hours
Outcome 7. Awakenings from sleep
Outcome 8. Breathing difficulties
Outcome 9. Daytime Sleepiness
Outcome 13. Movement ability – Gross motor function
Outcome 14. Manual ability (fine motor function)
Outcome 16. Ability to join activities with others
Outcome 18. Friendships
Outcome 19. Engagement in school life
Outcome 16. Ability to join activities with others
Outcome 17. Experience of other people's attitudes towards epilepsy
Outcome 22. Behavioural concerns
Outcome 23. Impulsivity
Outcome 25. Feelings about having epilepsy
Outcome 31. Self-esteem
Outcome 32. Mood swings
Outcome 34. Concealment
Outcome 36. Literacy
Outcome 37. Speech & Language
Outcome 41. School attendance
Outcome 42. Academic attainment
Outcome 43. Executive functioning
Outcome 46. Relationships with parents & siblings
Outcome 47. Family life
Outcome 48. Parental health
Outcome 51. Epilepsy specific attendance at A&E and/or unplanned admission to the ward

Total: 39

Voting Results:

Outcome 4 – Seizure Severity

Definition: How bad seizures are in terms of effects on the person during and after seizures – such as falls or injuries, incontinence, confusion and time to recover afterwards

Comments:

- AS (young person): 'Very important'

Votes in

Professionals: 9/9

Parents: 4/4

Young People: 2/2

Total: 15/15

Result: CONSENSUS IN

Outcome 5 – Time to fall asleep

Definition: Time it takes to fall asleep from snuggling down

Votes in

Professionals: 0/9

Parents: 0/9

Young people: 0/9

Total: 0/15

Result: CONSENSUS OUT

Outcome 6 – Time spent asleep in 24 hours

Definition: Total time spent asleep each day

Comments:

- AS (Young Person) – Time spent asleep not that important – not critical to research
- JH (Professional) – Sleep deprivation is a strong driver for seizures and probably severity
- RW (Professional) – Day and night should be split
- CM (Facilitator) – Can re define if we want to. Could change to 'total time spent asleep at night'

Vote on 'Time spent asleep each day'

Votes in

Professionals: 7/8 (BC didn't answer)

Parents: 4/4

Young People: 0/2

Total: 11/14

Result: CONSENSUS OUT

Vote on 'Time spent asleep in 24 hours': CONSENSUS IN
Vote on 'Time spent asleep at night': CONSENSUS IN

Outcome: Time spent asleep at night
Definition: Total time spent asleep at night

Votes in
Professionals: 8/8
Parents: 4/4
Young People: 2/2
Total: 14/14

Result: CONSENSUS IN

Outcome 7 – Awakenings from sleep
Definition: Waking in the night that parents/carers are aware of

Comments:

- JC (Parents): Critical

Votes in
Professionals: 9/9
Parents: 4/4
Young People: 2/2
Total: 15/15

Results: CONSENSUS IN

Outcome 8 – Breathing difficulties during sleep
Definition: Might include snoring or gasping for breath

Comments: PG (Professional) Important, epilepsy or not

First vote
Votes in
Professionals:7/9
Parents: 4/4
Young People: 0/2
Total: 11/15

Second vote
Votes in
Professionals: 9/9
Parents: 4/4
Young People: 2/2
Total: 14/15

Result: CONSENSUS IN

Outcome 9 – Daytime sleepiness
Definition: Feeling sleepy or actually sleeping during the day

Votes in:
Professionals: 9/9
Parents: 3/4
YP: 2/2
Total: 14/15

Results: CONSENSUS IN

Outcome 10 – Fatigue
Definition: Lacking in energy

Votes in:
Professionals: 0/9
Parents: 0/4
Young People: 0/2
Total: 0/15

Result: CONSENSUS OUT

Outcome 11 – Pain
Definition: Unpleasant, physical sensation

Comments:
- JC (Parent) – felt pain fitted in with severity of seizure

Professionals: 0/9
Parents: 0/4
Young People: 0/2
Total: 0/15

Result: CONSENSUS OUT

Outcome 13 – Movement ability – Gross motor function

***Combined Coordination & Balance outcome and definition.**

Definition: Using parts of the body together efficiently, such as to ride a bike, or stand on one leg, catching and throwing. Running, jumping, hopping, throwing.

Comments:

- AS (Young person) felt it was very important for school especially for moving around to different lessons.

Professionals: 9/9
Parents: 4/4
Youn People: 2/2
Total: 15/15

Result: CONSENSUS IN

Outcome 14 -Manual ability (fine motor function)

Definition: Dexterity (skill) in handling objects, handwriting

Comments:

- DR (Parent) thinks movement ability, coordination & balance, and manual ability are all the same.
- CT (Professional) Wondered if it was 'critical' to measure
- CT (Parent) In order to get support for a child, anything that can support an application for parents.

Vote in
Professionals: 8/9
Parents: 4/4
Young People: 2/2
Total: 14/15

Result: CONSENSUS IN

Outcome 15: Self care

Definition: Daily routines such as eating, washing, dressing and toileting.

Note: RW left the meeting. Total out of 14 professionals.

Professionals
Parents
Young People
Total

Results: CONSENSUS OUT

<u>Outcome 16:</u> Ability to join activities with others
Definition: Joining in with people such as playing out with friends, sleepovers, doing sports, joining in things
*Combined Social life with this outcome, added in the word 'sleepovers'.

Vote in (when combined)
Professionals: 8/8
Parents: 4/4
Young People: 2/2
Total:

Result: CONSENSUS IN

<u>Outcome 17:</u> Ability to play on one's own
Definition: Reading, computer games, imaginary play etc.

Professionals: 0/8
Parents: 0/4
Young People: 0/2
Total: 0/14

Result: CONSENSUS OUT

<u>Outcome 18:</u> Friendships
Definition: Forming and maintaining friendships

Vote in:
Professionals: 7/8
Parents: 4/4
Young People: 2/2
Total: 13/14

Result: CONSENSUS IN

<u>Outcome 19:</u> Engagement in school life
Definition: Feeling part of the school community

Vote in
Professionals: 8/8
Parents: 4/4
Young People: 2/2
Total: 14/14

Result: CONSENSUS IN

Outcome 21: Experience of other people's attitudes towards epilepsy
Definition: How people behave towards someone with epilepsy which could include things like bullying or social exclusion

Vote in
Professionals: 8/8
Parents: 4/4
Young People: 2/2
Total: 14/14

Result: CONSENSUS IN

Outcome 22: Behavioural Concerns
***Name changed from Behaving appropriately**
Definition: Being able to control emptions and respond to situations in context

Vote in
Professionals: 8/8
Parents: 4/4
Young People: 2/2
Total: 14/14

Result: CONSENSUS IN

Outcome 23: Impulsivity
Definition: Acting without thinking

Vote in
Professionals: 7/8
Parents: 3/4
Young People: 1/2
Total: 11/14

Result: CONSENSUS IN

Outcome 24: Fidgeting

Definition:

Vote in
Professionals: 0/8
Parents: 0/4
Young People: 0/2
Total: 0/14

Result: CONSENSUS OUT

Outcome 25: Feelings about having epilepsy
Definition: Feeling like other people of the same age
***combination of Feeling normal, Feelings about having epilepsy, Happiness, Sadness, Worried, Annoyed.**
Definition:

Vote in
Professionals: 8/8
Parents: 4/4
Young People: 2/2
Total: 14/14

Result: CONSENSUS IN

Outcome 31: Self esteem
Definition: Overall feelings about yourself

Vote in
Professionals: 8/8
Parents: 4/4
Young People: 2/2
Total: 14/14

Result: CONSENSUS IN
Outcome 32: Mood swings
Definition: Quick unexplained changes of mood

Vote in
Professionals: 8/8
Parents: 4/4
Young People: 2/2
Total: 14/14

Result: CONSENSUS IN

Outcome 34: Concealment
Definition: Quick unexplained changes of mood

Vote in
Professionals: 6/8
Parents: 4/4
Young People: 2/2
Total: 12/14

Result: CONSENSUS IN

Outcome 36: Literacy
Definition: Reading, writing, spelling

Vote in
Professionals: 8/8
Parents: 4/4
Young People: 2/2
Total: 14/14

Result: CONSENSUS IN

Outcome 37: Speech & language
Definition: Making yourself understood and understanding when spoken to

Vote in
Professionals: 7/8
Parents: 4/4
Young People: 2/2
Total: 13/14

Result: CONSENSUS IN

Outcome 41: School attendance
Definition: Turning up and engaging in school curriculum

Vote in
Professionals: 8/8
Parents: 4/4
Young People: 2/2
Total: 14/14

Result: CONSENSUS IN

<u>Outcome 42:</u> Academic attainment
Definition: Reaching potential through studying and completing assigned tasks and projects and advancing to next stage of education

Vote in
Professionals: 8/8
Parents: 4/4
Young People: 2/2
Total: 14/14

Result: CONSENSUS IN

<u>Outcome 43:</u> Executive functioning
Definition: The ability to plan and organise complex activities and set goals and manage your time. Executive functions help you manage life tasks such as organizing a trip, homework and school projects.

Vote in
Professionals: 8/8
Parents: 4/4
Young People: 2/2
Total: 14/14

Result: CONSENSUS IN

<u>Outcome 46:</u> Relationships with parents & siblings
Definition: Getting along well with and feeling close to other members of the family

Vote in
Professionals: 8/8
Parents: 4/4
Young People: 2/2
Total: 14/14

Result: CONSENSUS IN

<u>Outcome 47:</u> Family life
Definition: Impact of epilepsy on family life such as parent work opportunities or/leisure time

Vote in
Professionals: 8/8
Parents: 4/4
Young People: 2/2
Total: 14/14

Result: CONSENSUS IN

Outcome 48: Parental health
Definition: Parents physical and emotional health

Vote in
Professionals: 8/8
Parents: 4/4
Young People: 2/2
Total: 14/14

Result: CONSENSUS IN

Outcome 50: Epilepsy specific attendance at A&E and/or unplanned admission to the ward
Definition: unexpectedly need to be admitted to hospital

Vote in
Professionals: 8/8
Parents: 4/4
Young People: 2/2
Total: 14/14

Result: CONSENSUS IN
