# Supplementary material for: Core Health Outcomes in Childhood Epilepsy (CHOICE): Development of a core outcome set using systematic review methods and a Delphi survey consensus
Source: Epilepsia. 2019 Apr 25;60(5):857–71. doi: 10.1111/epi.14735 (PMC6563436; doi:10.1111/epi.14735)
Supplement: Supplementary file 7 [file EPI-60-857-s007.pdf]

CHOICE – Core Health Outcomes in Childhood Epilepsy
COS STAR Checklist

SECTION/TOPIC	ITEM No.	CHECKLIST ITEM	PAGE No. IN DOCUMENT
**TITLE/ABSTRACT**			
Title	1a	Identify in the title that the paper reports the development of a COS	1
Abstract	1b	Provide a structured summary	3
**INTRODUCTION**			
Background and objectives	2a	Describe the background and explain the rationale for developing the COS	5-7
	2b	Describe the specific objectives with reference to developing a COS	5-7
Scope	3a	Describe the health condition(s) and populations(s) covered by the COS	5-7, 9
	3b	Describe the intervention(s) covered by the COS	5-7, 9
	3c	Describe the setting(s) in which the COS is to be applied	5-7, 9
**METHODS**			
Protocol/Registry Entry	4	Indicate where the COS development protocol can be accessed, if available, and/or the study registration details	8
Participants	5	Describe the rationale for stakeholder groups involved in the COS development	5-8, 10-12, 14-15

		process, eligibility criteria for participants from each group, and a description of how the individuals involved were identified	
Information Sources	6a	Describe the information sources used to identify an initial list of outcomes	9-10, Figure 1, Supplementary 1 & 2
	6b	Describe how outcomes were dropped/combined, with reasons (if applicable)	9, Supplementary 2 & 4
Consensus Process	7	Describe how the consensus process was undertaken	10-12, 14-15 Supplementary 5
Outcome Scoring	8	Describe how the outcomes were scored and how scores were summarised	10-12, 14-15
Consensus Definition	9a	Describe the consensus definition	10-12, 14-15
	9b	Describe the procedure for determining how outcomes were included or excluded from consideration during the consensus process	10-12, 14-15 Table 4, Supplementary 4 and 5
Ethics and Consent	10	Provide a statement regarding the ethics and consent issues for the study	8
**RESULTS**			
Protocol Deviations	11	Describe any changes from the protocol (if applicable), with reasons, and describe what	10, 14-15

		impact these changes have on the results	
Participants	12	Present data on the number and relevant characteristics of the people involved at all stages of COS development	14-15,<br>Table 2 and Supplementary 2 & 5
Outcomes	13a	List all outcomes considered at the start of the consensus process	Figure 2
	13b	Describe any new outcomes introduced and any outcomes dropped, with reasons, during the consensus process	Table 4,<br>Supplementary 4 & 5
COS	14	List all the outcomes in the final COS	Figure 2
DISCUSSION			
Limitations	15	Discuss any limitations in the COS development process	15-21
Conclusions	16	Provide an interpretation of the final COS in the context of other evidence, and implications for future research.	17-21
OTHER INFORMATION			
Funding	17	Describe sources of funding/role of funders	21
Conflicts of interest	18	Describe any conflicts of interest within the study team and how these were managed	22
